# Spermatozoon-propelled microcellular submarines combining innate magnetic hyperthermia with derived nanotherapies for thrombolysis and ischemia mitigation

**DOI:** 10.1186/s12951-024-02716-w

**Published:** 2024-08-08

**Authors:** Pei-Wei Weng, Chia-Hung Liu, Pei-Ru Jheng, Chia-Che Chiang, Yan-Ting Chen, Lekshmi Rethi, Yves S. Y. Hsieh, Andrew E.-Y. Chuang

**Affiliations:** 1 Graduate Institute of Biomedical Materials and Tissue Engineering, International Ph.D. Program in Biomedical Engineering, Graduate Institute of Nanomedicine and Medical Engineering, College of Biomedical Engineering, Taipei Medical University, New Taipei City, 23561 Taiwan; 2 Department of Orthopedics, Shuang Ho Hospital, Taipei Medical University, New Taipei City, 23561 Taiwan; 3 Department of Orthopedics, School of Medicine, College of Medicine, Taipei Medical University, Taipei, 11031 Taiwan; 4 Department of Urology, School of Medicine, College of Medicine, Taipei Medical University, Taipei, 11031 Taiwan; 5 TMU Research Center of Urology and Kidney, Taipei Medical University, Taipei, 11031 Taiwan; 6 Department of Urology, Shuang Ho Hospital, Taipei Medical University, New Taipei City, 23561 Taiwan; 7 School of Pharmacy, College of Pharmacy, Taipei Medical University, Taipei, 11031 Taiwan; 8 Division of Glycoscience, Department of Chemistry, School of Engineering Sciences in Chemistry, Biotechnology, and Health, KTH Royal Institute of Technology, Alba Nova University Centre, Stockholm, SE106 91 Sweden; 9 Cell Physiology and Molecular Image Research Center, Taipei Medical University-Wan Fang Hospital, Taipei, 11696 Taiwan; 10 Precision Medicine and Translational Cancer Research Center, Taipei Medical University Hospital, Taipei, 11031 Taiwan

**Keywords:** Thrombolysis, Spermatozoon-propelled microcellular submarine, Innate magnetic hyperthermia, Exosome vesicle, Nanopayload, Ischemia mitigation

## Abstract

Thrombotic cardiovascular diseases are a prevalent factor contributing to both physical impairment and mortality. Thrombolysis and ischemic mitigation have emerged as leading contemporary therapeutic approaches for addressing the consequences of ischemic injury and reperfusion damage. Herein, an innovative cellular-cloaked spermatozoon-driven microcellular submarine (SPCS), comprised of multimodal motifs, was designed to integrate nano-assembly thrombolytics with an immunomodulatory ability derived from innate magnetic hyperthermia. Rheotaxis-based navigation was utilized to home to and cross the clot barrier, and finally accumulate in ischemic vascular organs, where the thrombolytic motif was “switched-on” by the action of thrombus magnetic red blood cell-driven magnetic hyperthermia. In a murine model, the SPCS system combining innate magnetic hyperthermia demonstrated the capacity to augment delivery efficacy, produce nanotherapeutic outcomes, exhibit potent thrombolytic activity, and ameliorate ischemic tissue damage. These findings underscore the multifaceted potential of our designed approach, offering both thrombolytic and ischemia-mitigating effects. Given its extended therapeutic effects and thrombus-targeting capability, this biocompatible SPCS system holds promise as an innovative therapeutic agent for enhancing efficacy and preventing risks after managing thrombosis.

**Graphical abstract:**

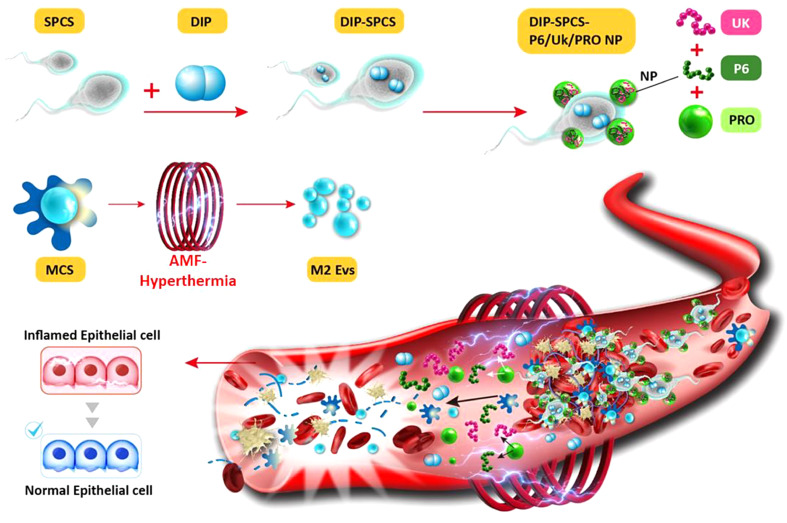

**Supplementary Information:**

The online version contains supplementary material available at 10.1186/s12951-024-02716-w.

## Introduction

Cardiovascular diseases remain a global health challenge, with thromboses being the primary cause of mortality among patients afflicted by these ailments [1, 2]. Thrombus formation typically arises due to disruptions in hemostasis regulation and is often referred to as “the silent killer,” since it can remain asymptomatic until it reaches an advanced stage. Thrombi within blood vessels can lead to conditions such as myocardial infarction, ischemic stroke, enduring disability, and even fatal consequences [3, 4]. Swift thrombus removal is crucial for the timely restoration of blood circulation, preventing severe complications and potentially saving lives. Regular patient healthcare often involves reperfusion therapy that employs thrombolytic agents and/or anticoagulants. Anticoagulants such as hirudin peptide (P6) and dipyridamole (DIP) are applied to treat mild thrombotic conditions [5]. These anticoagulants not only hinder the progression and penetration of thrombi but also act as deterrents against the formation of blood clots [6]. The systemic administration of thrombolytic agents, including tissue plasminogen activators (tPAs) and urokinase (Uk), can actively dissolve thrombi or initiate thrombolytic pathways [7]. It is important to note that these thrombolytic agents have relatively short half-lives; for instance, tPA has a half-life of approximately 4 ~ 6 min, and that of the Uk is around 10 ~ 15 min. Therefore, repeated high-dose administration is often required to achieve an effective thrombolytic response [8]. However, the poor biodistribution of thrombolytic agents and/or anticoagulants, along with their excessive use, can be detrimental to physiological hemostasis and often leads to complications of recurrence, side effects, and ischemic injury-derived neural or tissue damage. These issues significantly limit the available therapeutic timeframes and can substantially offset potential therapeutic advantages [9].

To tackle restrictions due to insufficient pharmacokinetics, thrombolytic drug-delivery systems are emerging to achieve targeted accumulation and controllable release at thrombus lesions. To augment circulatory bioavailability and stability, antithrombotic agents have been capsulated within liposomes, hydrogels, and microcapsules, or conjugated with poly(ethylene glycol) [10]. To mitigate the risk of bleeding, nanocarriers are modified with affinity moieties that target activated platelets and fibrin, examples of which include fucoidan and anti-fibrin peptides. These modifications enhance the retention of the drug at the intended site [11]. However, when encountering compact fibrin networks of blood clots, a significant challenge for these drug carriers is their limited ability to effectively penetrate thrombi, as they tend to stay primarily on the interface of thrombi [12]. Following clinical guidelines, it is recommended that antithrombotic agents be administered within 3 to 12 h after the onset of symptoms [13]. In contrast to passive diffusion, which relies on concentration-dependent mechanisms, drug carriers are now incorporating additional driving forces to create micromotors, which are being explored as a means of enhancing the penetration of therapeutic agents into targeted lesions to overcome physiological barriers [14].

In pursuing micromotor development for antithrombotic applications, it is essential to consider the shape as a critical factor influencing the lateral drift toward blood vessel walls. Various shapes, including tubular, spherical, and rod-shaped micromotors, have been proposed for this purpose [15]. A sphere-shaped structure has generally been studied, and a Janus structure is usually generated by grafting with enzymes (e.g., urease and catalase) or selective deposition of metal caps (e.g., platinum (Pt) and gold (Au)). Catalytic chemical reactions provide a driving force to move particles in a unidirectional, non-reciprocating manner [16]. In contrast to the streamlined movement of spherical counterparts, non-spherical particles exhibit rotation, torque, and tumbling in the systemic circulation. This dynamic behavior enhances their interactions with clots on vessel walls [16]. Moreover, non-spherical particles have demonstrated extended systemic circulation compared to their spherical counterparts and are subject to lower uptake by immune cells. This characteristic facilitates increased drug accumulation at thrombus sites [17]. Compared to the propulsion of nanorods or Janus nanospheres from one side, the tubular shape was proven to be more advantageous for achieving swift motion through efficient bubble injection from the opening of the tube [18].

An essential consideration in micromotor design is the incorporation of a propulsive force from microtubes. At present, the utilization of metal caps on spherical micromotors and metal layers inside microtubes generates gas bubbles once exposed to gastric acid or in the presence of hydrogen peroxide. However, this approach is biologically incompatible with uses involving systemic circulation [19]. External forces, such as magnetic fields and light irradiation, have been harnessed to enhance thrombolytic effectiveness. Wang et al. devised mesoporous Pt/silica motors intended for anticoagulant medications and targeted delivery for thrombolytic use. The motion competences of those motors, when subjected to near-infrared (NIR) irradiation, proved greatly effectual in retaining medicines in thrombi and facilitating deep infiltration [12]. Xie et al. engineered micromotors with entrenched, well-organized magnetic nanoparticle chains enclosed within microgel shells. These micromotors offered the capability to regulate the delivery and release of thrombolytic drugs through the use of a magnetic field [20]. However, such treatments often necessitate the expertise of trained professionals and specialized equipment. Additionally, the depth to which light irradiation can penetrate is relatively shallow [21]. In contrast, biological cells are harnessed to induce motion through their inherent cellular behaviors, eliminating the need for additional fuels and external fields. Cells like spermatozoa, with their natural swimming ability owing to the existence of microtubular flagella and rheotaxis abilities, can be employed to generate directed motion toward highly viscous thrombi and penetration into a clot [22]. Nonetheless, there is no documented instance of antithrombotic interventions being aided by spermatozoon-propelled motors.

Thrombus formation is characterized by the accumulation of resident blood cells such as red blood cells (RBCs), platelets, and macrophages, which in turn causes a rise in thrombus viscosity [23–26]. RBCs containing deoxygenated reduced ferric ions (Fe^2+^) exhibit innate magnetic responsiveness [27]. Magnetic hyperthermia is a remote non-invasive technique that employs magnetic materials, such as iron oxide nanoparticles, to generate heat when subjected to an alternating magnetic field (AMF). A prior study showed that utilizing magnetic hyperthermia on blood clots can improve the thrombolysis of contracted blood clots and potentially extend the therapeutic window for effective thrombolysis [28]. Nonetheless, findings from a previous study indicated that the consumption of iron materials in connection with orthopedic implants should be considered a potential risk factor for neurological disorders [29]. Naturally, biocompatible and magnetically responsive RBCs offer a unique and innovative alternative to replacing iron materials in the context of magnetic hyperthermia for thrombus management. Contemporary research suggests that heat stress from hyperthermia can trigger a protective effect from heat shock proteins (HSPs) and facilitate macrophages to secrete extracellular vesicles (EVs) [30]. Recent studies revealed that periodic mild hyperthermia can steer macrophages toward the anti-inflammatory M2 phenotype and promote expressions of anti-inflammatory cytokines [31].

Herein, we put forward a strategy for infiltrating and managing blood clots utilizing spermatozoon-propelled cellular submarines (SPCSs). SPCSs were designed to deliver Uk and DIP, aiming for enhanced penetration into blood clots and achieving effective thrombolysis. DIP was first diffused into SPCSs to create DIP-SPCSs. The improvement persisted with the development of P6/Uk NPs that were engineered to lade Uk and P6 onto DIP-SPCSs. NPs were prepared by protamine (PRO) incorporating P6 and Uk as a template, and the formed P6/Uk/PRO NPs were assembled onto DIP-SPCSs to construct microtubular motors. This was cleverly coupled with P6/Uk/PRO NPs to create remarkable DIP-SPCS-P6/Uk/PRO NP micromotors. This complex coordination is clearly shown in Graphical abstract, where these micromotors, propelled by the remarkable force of spermatozoon propulsion, precisely used rheotaxis-guided navigation to find and pierce blood clots.

As depicted in Graphical abstract, the DIP-SPCS-P6/Uk/PRO NPs could navigate toward blood clots by utilizing rheotaxis-based guidance and subsequently penetrate clots by employing flagellum propulsion. In addition, to augment the synergistic effects of DIP-SPCS-P6/Uk/PRO NPs, thrombus RBCs induce magnetic hyperthermia upon application of an AMF. This, in turn, triggers the release of therapeutics from the SPCS carrier and the production of EVs from resident polarized M2 macrophages (MCS EVs). These events contributed to the immunomodulatory, anti-inflammatory, and neuroprotective effects. This cascade of actions resulted in a remarkable synergy between innate magnetic hyperthermia, sperm propulsion, and the delivery of thrombolytic therapeutics, ultimately leading to a substantial reduction in the risk of ischemic injury and recurrence. Moreover, it significantly enhanced the efficiency of therapeutic delivery and thrombolysis.

**Scheme 1:**
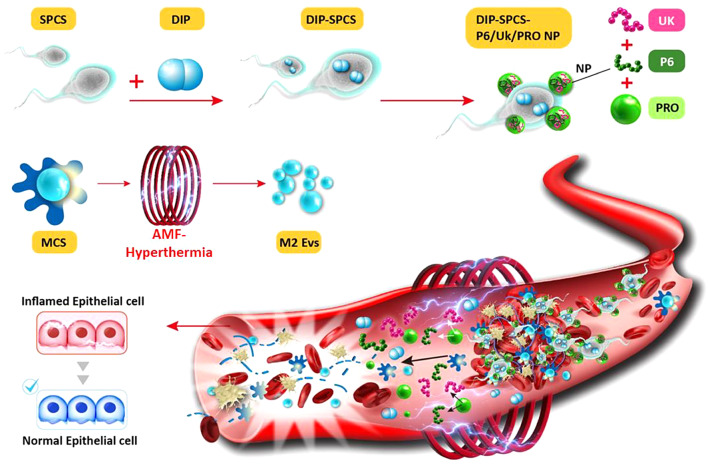
Diagram illustrating the step-by-step process for fabricating dipyridamole (DIP)-spermatozoon-propelled cellular submarine (SPCS)-hirudin peptide (P6)/urokinase (Uk)/protamine (PRO) NPs and depicts their thrombolytic mechanism. DIP was diffused into SPCSs to form DIP-SPCSs. P6/Uk/PRO NPs were subsequently created through an ionic self-assembly process. The P6/Uk/PRO NPs adhered to DIP-SPCSs via electrodeposited interactions, resulting in the assembly of DIP-SPCS-P6/Uk/PRO NPs. These DIP-SPCS-P6/Uk/PRO NPs then employed rheotaxis-driven motion to effectively penetrate thrombi. To augment the effectiveness of DIP-SPCS-P6/Uk/PRO NPs, thrombus red blood cells (RBCs) were induced to undergo magnetic hyperthermia when subjected to an alternating magnetic field (AMF). This magnetic hyperthermia facilitated the release of therapeutics from the SPCS carrier and the production of extracellular vesicles by innate M2 macrophages (MCS EVs). This combined action possessed immunomodulatory, anti-inflammatory, and neuroprotective advantages. The synergy achieved through this approach substantially mitigated the risk of ischemic injury and recurrence while simultaneously elevating the efficiency of therapeutic delivery and thrombolysis

## Materials and methods

### Materials

DIP, Uk, propidium iodide (PI), diacetyldichlorofluorescein (DCFH-DA), 4,6-diamidino-2-phenylindole (DAPI), and lipopolysaccharide (LPS) were commercially obtained from Sigma-Aldrich (St. Louis, MO, USA). A mouse macrophage marker (F4/80), cyanine 5 N-hydroxy succinimide ester (Cy5-NHS ester), anti-HSP70, anti-M2 macrophage (CD206), and anti-EV (CD81) were purchased from Asia Bioscience (Taipei, Taiwan). A Live/Dead kit was from ThermoFisher Scientific (Waltham, MA, USA). We utilized the C-terminal peptide fragment technique to synthesize fluorescent CR110-conjugated P6 [26]. The mass spectrometric experiment verified the molecular weight of the synthesized P6 compound (Fig. S1). Furthermore, covalent conjugation was used to produce the fluorescent Cy5 NHS ester-labeled protein, Uk, following our earlier research [26].

Male ICR mice at 6 − 8 weeks of age were obtained from BioLASCO (Taipei, Taiwan). The Taipei Medical University’s animal facility housed animals under protocols approved by the university’s animal care committee, and animals were permitted unlimited access to food and water. SPCSs were isolated from epi-testinal tissues of male ICR mice using a SpermGrad™ (Virtolife, Englewood, CO, USA) kit through gradient buffer purification at pH 7.4 and 37 °C in 1× phosphate-buffered saline (PBS) under sterile centrifugation (for 20 min at 300 − 600 *g*), as per a instruction procedure.

### Experimental section

#### P6/Uk/PRO NP and DIP-SPCS-P6/Uk/PRO NP preparation and characterization

SPCSs (at a concentration of 10^6^ cells/mL at 37 °C and pH 7.4) were dispersed in varying DIP concentrations (0 − 1 mg/mL in 1× PBS at pH 7.4 and 37 °C) to load DIP into SPCSs (DIP-SPCSs) via cell membrane permeation. Centrifugation was carried out at 1000 rpm for 10 min after dispersion to remove the supernatant that contained unloaded DIP. Purified DIP-SPCSs were gently resuspended in 1× PBS (at pH 7.4 and 37 °C). A fluorescence spectrometer (Leica Biosystems, Wetzlar, Germany) was used to quantify the resultant pellet for cellular DIP encapsulation. This assay evaluated the amount of DIP encapsulated in SPCS cells.

Different ratios of PRO to Uk and P6 were utilized to produce P6/Uk/PRO NPs. Mass ratios of P6/Uk/PRO were 1/1/5, 1/1/10, 1/1/20, 1/1/40, and 1/1/80 to obtain optimized NP formations, which involved stirring the mixture at room temperature for 20 min. Dynamic light scattering (DLS ZS90, Panalytical, Malvern, UK) was utilized to evaluate the size and surface charge of the formulated P6/Uk/PRO NPs, and transmission electron microscopy (TEM HT-7700, Hitachi, Tokyo, Japan) was employed to investigate micromorphological changes in P6/Uk/PRO NPs. Surface charges of DIP-SPCS-P6/Uk/PRO NPs were assessed under different feeding amounts of P6/Uk/PRO NPs, which were combined with DIP-SPCSs at a cell concentration of 10^6^ cells/mL.

The efficiency of the drug-encapsulation process was evaluated through an assessment of the encapsulation efficiency. To this end, CR 110-P6/Cy5-Uk/PRO NPs containing CR 110-P6, Cy5-Uk, and PRO, encapsulated within DIP-SPCSs, were synthesized at a density of 10^6^ sperm/mL, utilizing sperm medium (SP-TALP). Following the sperm’s exposure to the nanoparticle solutions, the resulting supernatant was collected post-centrifugation at 1000 rpm for 10 min and subsequently filtered via a membrane with a pore size of 2 μm [32]. Post-purification, the sperm were resuspended. Concentrations of DIP, CR 110-P6, and Cy5-Uk in these samples were ascertained with a fluorescence spectrometer by Leica Biosystems, which also facilitated the calculation of the total drug mass. Quantification of residual concentrations of DIP (with excitation λ at 380 nm and emission λ at 430 nm), CR 110-P6 (with excitation λ at 499 nm and emission λ at 525 nm), and Cy5-Uk (with excitation λ at 651 nm and emission λ at 670 nm) involved analyzing each sample’s supernatant post-centrifugation. The encapsulation efficiency was determined by the proportion of the encapsulated drugs (DIP, CR 110-P6, and Cy5-Uk) relative to the total quantity of drugs initially utilized, following the formula: Encapsulation Efficiency = [Total Drug Weight - Residual Drug Weight] / Total Drug Weight.

Furthermore, the quantity of drug loading was ascertained by comparing the initial drug amounts before incubation against the leftover quantities in the supernatant post-incubation, with quantification achieved through their respective fluorescence markers. Stability tests for encapsulation were conducted by monitoring the cumulative drug (DIP, CR 110-P6, and Cy5-Uk) release ratio from sperm into SP-TALP-containing mouse blood (treated with 3.8% sodium citrate), under conditions both with and without an AMF (9 V, 5 min). Specifically, drug-laden sperm were incubated in SP-TALP under dark, humidified conditions, with a 5% CO_2_ atmosphere at 37 °C. At predetermined intervals, 0.5 mL of supernatant was collected following centrifugation and replenished with fresh SP-TALP. Following a 24-h period, an EDTA-trypsin treatment lasting 3 min facilitated the complete release of drugs into the solution, with the SpectraMax M2 (Molecular Devices) fluorescence spectrometer employed in Leica Biosystems to measure drug concentrations (DIP, CR 110-P6, and Cy5-Uk) in the supernatant. The cumulative release ratio was calculated by dividing the accumulated drug amount by the total initial drug amount. This experiment was replicated across three samples, with free drugs (DIP, CR 110-P6, and Cy5-Uk) in SP-TALP serving as a control.

Using a fluorescence microscope (Leica Biosystems), the biodistribution of DIP within DIP-SPCS-P6/Uk/PRO NPs was observed. SPCS viability was evaluated with a live/dead assay of cultured SPCS cells. The staining solution was made by adding 5 µL of calcein AM (component A) and 20 µL of ethidium homodimer-1 (component B) to 10 mL of DPBS. The staining solution (100–200 µL) was directly added to cells after removing the medium. Cells were incubated at 20–25 °C for 30 min and then underwent a fluorescent microscopic analysis.

The size distribution of the generated cellular EVs (Nano Sight NS300 with a camera type of scalable complementary metal-oxide semiconductor (sCMOS), Malvern Panalytical) [33] was characterized using a nanotracking analysis (NTA, NanoSight NS300, Malvern Panalytical). Formulations were quantified using SEM and an energy-dispersive (EDS) x-ray analysis (SU3500 Hitachi, Tokyo, Japan), Fourier transformation infrared (FTIR) spectroscopy (Nicolet™ iS™ 10, ThermoFisher Scientific, with a 400–4000 cm^− 1^ spectral range), and 1 H-proton nuclear magnetic resonance (NMR) at room temperature with 0.15-ppm resolution at 300 °K [34] (Agilent 600 MHz DD2 NMR, Agilent Technologies, Santa Clara, CA, USA). A Bruker-AXS D2 Phaser (ST, USA) in Bragg-Brentano geometry was used to perform x-ray diffraction (XRD) measurements. A 2θ range from 10° to 60° with a step size of 0.016° was used.

#### Clot penetration and in vitro experiments

To generate in vitro blood clots, inflammation was induced in ICR mice through an LPS pre-injection (5 mg/kg body weight (BW)). Subsequently, whole blood was drawn into a 3.2% sodium citrate tube and centrifuged for 10 min at 600 *g* to extract the supernatant, and 70 µL of serum was combined with 10^8^ RBCs/mL, 10^8^ white blood cells (WBCs)/mL, and 10^8^ platelets/mL to form a final concentration of 1 U/mL thrombin and 2.5 mM CaCl_2_.

In vitro experiments were carried out to assess whether the SPCSs possessed clot-targeting and -penetrating abilities by (A) adding 5 × 10^5^ live or dead SPCS cells/mL (0.2 mL, in 1× PBS at pH 7.4 and 37 °C) (dead SPCSs were induced using a 5% dimethyl sulfoxide (DMSO) method) around a preformed clot in a glass-bottom culture dish (2D), or (B) using a transwell system with a clot in the lower chamber and live or dead SPCSs in the upper insert (with a pore size of 8 μm). The cell concentration was 5 × 10^5^ cells/mL (0.2 mL). Using an optical microscope (Leica), the targeting and penetration behaviors of SPCSs into the clot were observed after incubation for designated times [35–37].

To evaluate the magnetic hyperthermic performance, clots were formed in capillary tubes and subjected to an AMF (9 V), and temperature changes were monitored with a thermocouple. Control groups included PBS and unclotted blood samples. To evaluate the efficacy of magnetic hyperthermia, blood clots were artificially created within capillary tubes and subsequently subjected to an AMF of 9 V. In this assessment, microcentrifuge tubes contained 0.5 mL of PBS, 0.5 mL of mouse whole blood (obtained via cardiac puncture and pre-added to an anticoagulant tube), and 0.5 mL of clots. Subsequently, samples were introduced to the AMF (9 V), and subsequent temperature changes were observed and recorded for 5 min.

The impact of hyperthermia on macrophage polarization towards M2 EVs was assessed by incubating RAW 264.7 cells at 37–45 °C in 1× PBS (pH 7.4). EV (CD81, 1:100, 37 °C, 1 h) and M2 (CD206, 1:100, 37 °C, 1 h) levels were analyzed by flow cytometry (BD FACSVia™ Flow Cytometry System, BD Biosciences, San Jose, CA, USA), and EV numbers were measured with an NTA (NanoSight NS300, Malvern Panalytical).

To assess the effectiveness of DIP-SPCS-P6/Uk/PRO NPs in interacting with blood clots, a comprehensive experiment was conducted. First, a sample of FITC-fibrinogen was prepared to facilitate easy visualization and analysis under a fluorescent microscope [38]. The preparation process involved mixing fibrinogen with FITC in PBS for 4 h, followed by dialysis to remove any unbound FITC.

Next, fibrin clots were formed on a small Petri dish, after which a mixture of fibrinogen, 10^8^ cells/mL RBCs, 10^8^ cells/mL platelets, 10^8^ cells/mL WBCs, 20 µL of CaCl_2_ (0.2 mg/mL), and 2 µL of thrombin (100 U/mL) were added and incubated at 37 ℃. This step was created to replicate the conditions of a blood clot in a controlled environment. After the incubation period, the dishes were randomly grouped and subjected to testing with various substances, including Uk, P6, DIP, Uk + P6 + DIP, AMF, SPCS, DIP-SPCS-P6/Uk/PRO NPs + AMF, or DIP-SPCS-P6/Uk/PRO NPs. To ensure accuracy, the dishes were washed three times with PBS before being observed under a fluorescence microscope.

Blood freshly sourced from the white rabbits was treated with heparin to ensure stability. An 8-mL sample of this venous blood was then carefully collected into a sterilized tube. This preparatory step was for the subsequent blood-compatibility evaluation. To isolate intact RBCs from the rabbit blood for testing, the sample was centrifuged at 4 °C and 3500 rpm, effectively allowing the RBCs to precipitate.

Following separation, the precipitated RBCs were then diluted in a PBS solution and amplified tenfold in volume, to prepare them for the compatibility assay. Subsequently, 0.3 mL of this RBC solution was carefully combined with 1.2 mL of a specially formulated mixture containing DIP-SPCS-P6/Uk/PRO NP aggregates suspended in PBS, with deionized (DI) water serving as a positive control for the experiment. This blend was then subjected to further centrifugation at 3000 rpm. Post-centrifugation, the level of hemoglobin (HGB) released into the PBS supernatant was quantified using a microplate reader across a wavelength range of 400–900 nm, which served as a proxy for assessing blood compatibility [39].

### In vivo studies

The breeding of animals and the protocol were approved by the Institutional Animal Care and Use Committee of Taipei Medical University. ICR mice were anesthetized using isoflurane inhalation at a concentration of approximately 1–4%. Subsequently, depilatory cream was administered to the abdominal region to remove fur. An incision was made along the midline of the abdomen, allowing the mesenteric vessels to be exposed through a careful, non-cutting dissection technique.

To create the mesenteric vascular thrombosis model, 10 min of anesthesia with 1–4% isoflurane in oxygen was administered to ICR mice. The mesenteric vessels were then exposed and observed by opening the peritoneal cavity. The exposed mesenteric arteries were covered for 2 min with filter paper dipped in a 35% FeCl_3_ solution, and then again for 5 min. To assess the formulation’s ability to target vascular thromboses, mice were given either DIP + P6 + Uk or DIP-SPCS-P6/Uk/PRO NPs, which had equivalent doses of DIP, Uk, and P6. Using an in vivo imaging system (IVIS), DIP, CR110-P6, and cy5-Uk fluorescence signals were recorded after administration. After thrombus formation, DIP + P6 + Uk or DIP-SPCS-P6/Uk/PRO NPs (corresponding to equivalent doses to DIP, Uk, and P6) were given to evaluate the therapeutic effects in the mesenteric vascular thrombosis model. ImageJ software was used to perform the optical analysis and measure the fluorescence intensity. IVIS and fluorescence microscopic techniques were employed to quantitatively assess and visually depict the in vivo biodistribution patterns of DIP + P6 + Uk and SPCS-P6/Uk/PRO NPs. In brief, a volume of 100 µL of an aqueous DIP solution, with equivalent doses of DIP, Uk, and P6, or SPCS-P6/Uk/PRO NPs, was gradually administered to mice. The primary internal organs (spleen, lungs, heart, kidneys, and liver) of the animals that underwent AMF application were extracted. Tissues were first subjected to fixation by immersion in a solution of 10% formalin, followed by embedding in paraffin blocks and subsequent sectioning into approximately 8-µm-thick slices.

To assess the in vivo biodistribution of drugs fluorescent signals at varying time intervals (specifically 1, 5, and 14 days), animals were humanely euthanized, and principal organs (heart, liver, lungs, spleen, and kidneys) were collected for analysis. Subsequently, fluorescence images of the animals were acquired utilizing an IVIS (Lumina III XRMS) or fluorescence microscopy (Leica Biosystems) to observe the in vivo biodistribution. In addition, hematoxylin and eosin (H&E; Sigma, St. Louis, MO, USA) staining was performed to evaluate the histological morphology of treated vessels.

Immunofluorescence (IF) staining was performed on mesenteric tissues using the following antibodies and probes: F4/80 (diluted 1:1000, incubated for 2 h), PI, DCFH-DA (at a concentration of 40 µM, incubated for 30 min), anti-HSP70 antibody (diluted 1:100, incubated for 1 h), cluster of differentiation 206 (CD206; diluted 1:100, incubated for 1 h), and CD81 (diluted 1:100, incubated for 1 h). Furthermore, H&E staining and IF staining of F4/80, PI, DCFH-DA, HSP70, CD206, and CD81 were applied to tissues. Hematological parameters and biochemical markers, including RBCs, HGB, hematocrit (HCT), mean corpuscular volume (MCV), and white blood cells (WBCs), were evaluated using a Procyte Dx hematological analyzer from IDEXX Laboratories. This comprehensive analysis facilitated the detailed examination of blood serum to discern various health indicators. The utilization of the Procyte Dx analyzer enabled the precise measurement of the aforementioned hematological and biochemical indices, crucial for assessing the overall health and function of the blood. This advanced technology offers insights into the cellular composition and quality of the blood, providing a foundation for diagnosing potential disorders or monitoring the health status of subjects.

Ultrasonic (US) assessments were used to evaluate blood flow, and thermographic assessments were used to determine the vascular temperature. After applying the US couplant to tissues, a Doppler US flow detector (Philips) was to assess the velocity of blood flow at the vascular site. Mice were anesthetized using isoflurane and subsequently positioned beneath a US device for analysis. Blood flow was evaluated utilizing a color Doppler US device (Envisor, Philips). The blood flow velocity in the vessels of mice was quantified. The target tissue/area was coated with conductive gel, and the US probe was positioned above the blood vessels, ensuring proper contact with the conductive gel. The detection angle was modified until a consistent measurement of blood flow velocity was achieved.

To evaluate the general neuroprotective effect of the formulation in mice, behavioral tests such as a water maze analysis and gait analysis were performed [40, 41]. In this investigation, the assessment of gait spatiotemporal metrics was facilitated by a specialized walking track, enhanced with a video analysis system. The apparatus comprised an 80-cm-long, 6-cm-wide, and 12-cm-high Plexiglas enclosure. Below this structure, a mirror positioned at a 45° angle enabled the comprehensive observation and documentation using a high-speed digital camera (EX-F1, Casio, Iruma City, Japan). Before initiating the experimental trials, rats were acclimatized to the environment of the walking track by permitting unrestricted movement along it for 20 min. This preparatory step ensured that each rat was adequately familiar with the setup, aiming to minimize stress and potential variability in walking patterns. The walking exercise was conducted multiple times, specifically five or six iterations in each direction, with only the sequences comprising a minimum of four uninterrupted steps being documented. The digital recordings obtained from these trials were analyzed using MATLAB (MathWorks, vers. 7.6, R2008a, Natick, MA, USA) to facilitate threshold-based identification of hindlimb stepping patterns. From these analyses, four spatial parameters of stride frequency were derived and subsequently averaged over a minimum of 20 footstep instances.

In conjunction with the gait analysis, the mice were subjected to a preparatory phase involving 3 days of training in a Morris water maze, in anticipation of subsequent memory and learning evaluation exercises. The experimental arrangement for the Morris water maze consisted of a circular pool, measuring 100 cm in diameter and 50 cm in height, equipped with a submerged platform 9 cm in diameter. The task was monitored using a computerized tracking system, designed to record various performance metrics including the trajectory and time taken for each mouse to locate the platform. The water in the maze was maintained at a warm temperature, specifically 23–25 °C, with the water level set to challenge the mice’s spatial navigation skills. Consistency in the laboratory’s environmental conditions was strictly observed to mitigate extraneous variables.

### Statistical analysis

The mean ± standard deviation (SD) of experimental results is presented. The 2-way ANOVA was used to determine statistical significance, as indicated by * *p* < 0.0332, ** *p* < 0.0021, *** *p* < 0.0002, and **** *p* < 0.0001.

## Results and discussion

### Preparation of DIP-SPCS-P6/Uk/PRO NPs

**Fig. 1:**
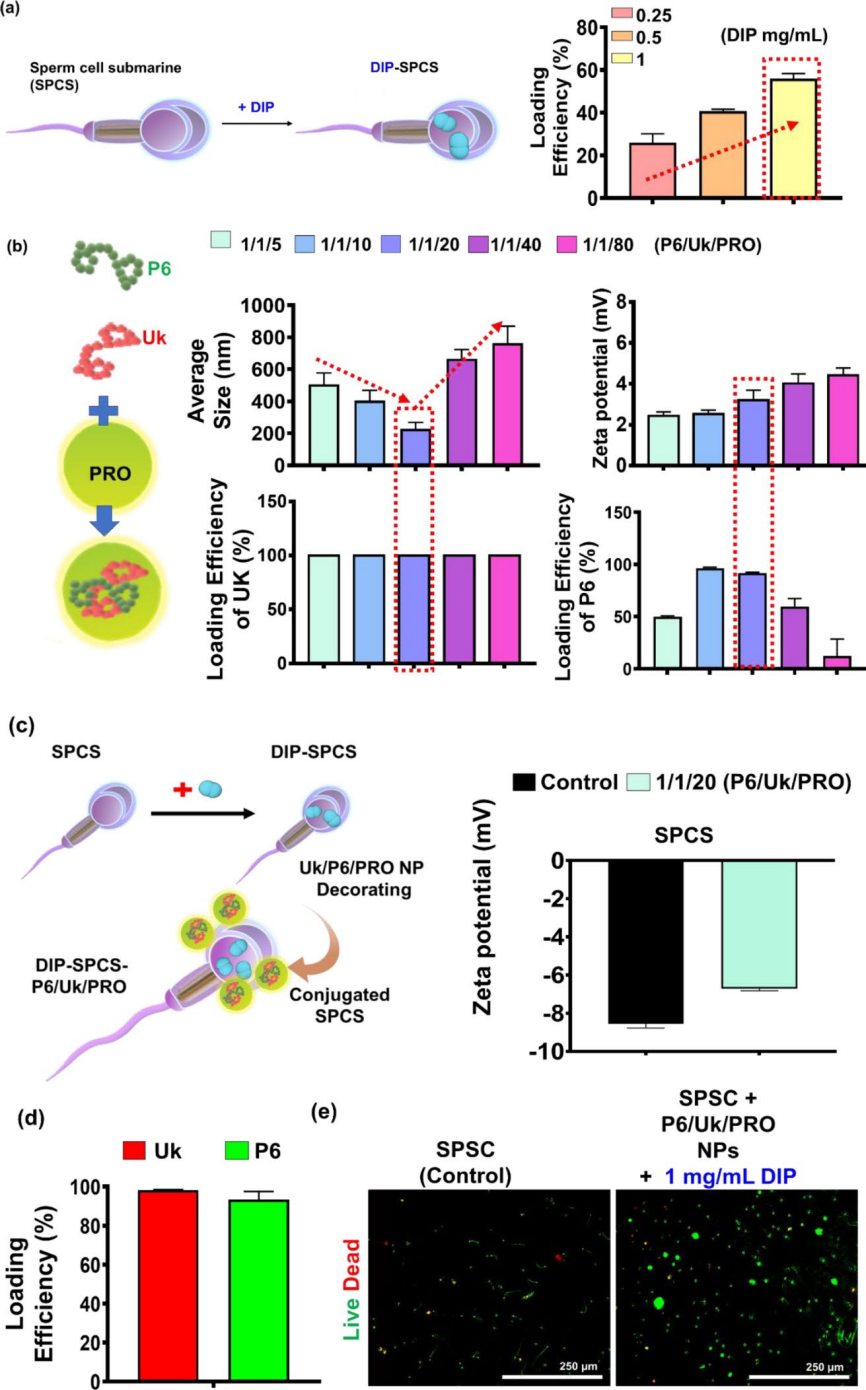
Preparation of dipyridamole (DIP)-spermatozoon-propelled cellular submarines (SPCSs) and DIP-SPCS-hirudin peptide (P6)/urokinase (Uk)/protamine (PRO) NPs is detailed as follows. (**a**) Depicted schematically is the formation of DIP-SPCSs through diffusion. Assessment of the DIP loading efficiency in SPCSs with varying DIP concentrations. The loading efficiency increased with higher DIP concentrations, indicating effective DIP loading via diffusion. Despite DIP’s hydrophilicity and molecular weight (505 g/mole), the loading efficiency was constrained to ca. 55%. (**b**) A schematic illustration outlines the creation of P6/Uk/PRO NPs via ionic gelation, with optimization based on the particle size, zeta potential, and drug-loading efficiencies determined using DLS and spectrometric methods. The optimal ratio (1/1/20) produced tightly packed nanoparticles averaging 228 nm, demonstrating enhanced formation and stability. Optimal formulation (1/1/20) yielded nanoparticles with a slight positive surface charge, enhancing cellular adherence and achieving high drug loading efficiencies for Uk (ca. 99%) and P6 (ca. 91%). (**c**) A schematic depiction illustrates the formation of DIP-SPCS-P6/Uk/PRO NPs through ionic interactions, with zeta potential data showing surface charge comparisons between DIP-SPCSs and DIP-SPCS-P6/Uk/PRO NPs. Successful assembly of DIP-SPCS-P6/Uk/PRO NP complexes was evidenced by the attraction and binding of positively charged NPs to negatively charged cell surfaces, supported by zeta potential measurements. (**d**) Spectrometric data demonstrate drug recovery following incorporation into DIP-SPCS-P6/Uk/PRO NPs. Loading percentages were ca. 98% for Uk and 93% for P6. (**e**) Fluorescence microscopic data show that SPCSs, after loading with drugs, maintained cell viability. Green fluorescence, indicative of living cells, predominated, confirming the SPCS carrier system’s ability to integrate P6/Uk/PRO NPs and encapsulate DIP without compromising sperm cell viability. Experimental results are presented as the mean ± SD

Aqueous DIP solutions at final concentrations of 0, 0.25, 0.5, and 1 mg/mL were cocultured with SPCS cells at a concentration of 10^6^ cells/mL in DIP-loading experiments (Fig. 1a). The study showed that the loading efficiency within SPCSs rose as the concentration of DIP increased, indicating the effectiveness of DIP loading through diffusion. DIP, with a molecular weight of 505 g/mole, is a hydrophilic medication that can traverse cell membranes due to its molecular weight being below the claimed limit of 1000 Da for hydrophilic drug diffusion [42]. Nevertheless, the loading efficiency into SPCS cells was only ca. 55% as a result of the solubility constraint of DIP, which is around 1 mg/mL (Fig. 1a). In the following experiments, a concentration of 1 mg/mL DIP was chosen for loading into SPCSs (DIP-SPCSs).

The nanocarrier, crosslinked with the FDA-approved positively charged PRO, can facilitate the incorporation of larger therapeutic molecules such as the fibrinolytic agent, Uk, and the antithrombotic agent, P6. Previous study provides compelling evidence of the technique’s capacity to nano-encapsulate hydrophilic medicines. Remarkably, this technique possesses the adaptability to incorporate challenging compounds within a polymeric framework, hence expanding its applicability in drug-delivery systems and unveiling novel avenues for research [43].

In the formulation experiment, the P6/Uk/PRO NP formulation was systematically varied with weight ratios of 1/1/5, 1/1/10, 1/1/20, 1/1/40, and 1/1/80 (mg) to investigate the effect of PRO on ionic gelation with P6 and Uk in equal measures. The objective was to optimize the composition for enhanced nanoparticle formation and stability. The experimental findings revealed that at the lower feed ratios of 1/1/5 and 1/1/10, the resultant nanoparticles exhibited sizes ranging from ca. 400–500 nm, as shown in Fig. 1b. The variation in size was caused by insufficient PRO, which did not fully connect with the negatively charged P6 and Uk. As a result, the particles had greater hydraulic diameters and a less tightly structured structure.

On the other hand, when the ratio of P6/Uk/PRO reached 1/1/20, the amount of PRO was determined to be optimal for facilitating sufficient ionic interactions between the components, resulting in the creation of nanoparticle complexes that are more tightly packed. The size of these complexes was greatly reduced, with an average of approximately 228 nm. However, when the PRO concentration was further increased, especially at feed ratios of 1/1/40 and 1/1/80, particle aggregation occurred because of the excessive amount of PRO. This resulted in the formation of bigger nanoparticles, measuring roughly 600 ~ 800 nm in size. Notably, the nanoparticles formulated with a 1/1/20 ratio also demonstrated a slight positive surface charge (Fig. 1b), which is beneficial for cellular adherence as suggested by previous studies. This optimal ratio yielded the most efficient drug loading capacities among the formulations tested, with Uk and P6 achieving respective loading efficiencies of ca. 99% and 91% (Fig. 1b, bottom). An in-depth comparative analysis across the different P6/Uk/PRO weight ratios highlighted the critical role of PRO in modulating the nanoparticle size and structure through ionic gelation mechanisms [44]. The study emphasized the balance required between ensuring adequate ionic interactions for stable nanoparticle formation and avoiding excessive PRO concentrations that lead to aggregation. The positive surface charge observed at the optimal ratio further underscored the importance of electrostatic interactions in facilitating the attachment of nanoparticles to cellular membranes, enhancing the potential for cellular uptake. The findings from this study not only shed light on the intricate dynamics of nanoparticle formation and stability but also established a foundation for future investigations into the precise engineering of nanoparticle systems for targeted drug delivery applications. The detailed quantitative comparisons of nanoparticle sizes, loading efficiencies, and surface charges across varying formulations provide valuable insights into the design of more effective and efficient nanoparticle-based therapeutics.

In the drug-loading study, nanoparticles composed of P6, Uk, and PRO (P6/Uk/PRO NPs) were prepared in a specific ratio of 1:1:20 and subsequently introduced to a culture containing DIP-SPCSs (10^6^ cells). The successful assembly of DIP-SPCS-P6/Uk/PRO NP complexes was evidenced by the attraction and binding of positively charged P6/Uk/PRO NPs to the negatively charged surfaces of cells, a process mediated by electrostatic forces. This interaction was quantitatively supported by zeta potential measurements, indicating a slight increase in the cell surface potential due to nanoparticle attachment, as illustrated in Fig. 1c.

To assess the effectiveness of incorporating the components of P6/Uk/PRO NPs into DIP-SPCSs, a centrifugation procedure was utilized. The results showed exceptional loading efficiency and recovery rates for DIP, Uk, and P6 in the DIP-SPCS-P6/Uk/PRO NP complexes. The loading percentages were ca. 98% for Uk, and 93% for P6 as described in Fig. 1d. To elaborate further, a comprehensive comparison analysis was carried out to examine the specific efficiencies of combining DIP, Uk, and P6, as well as their rates of recovery after the coculturing procedure. The objective of this investigation was to enhance our comprehension of the dynamics of interaction between the formulation and cell surfaces, emphasizing the exceptional accuracy with which these materials can be guided for cellular attachment. The high loading efficiencies of Uk and P6 indicate strong compatibility and excellent binding affinity of the nanoparticles towards DIP-SPCSs, highlighting the promise of this technique for targeted distribution applications. Twenty-four hours following the incorporation of the drug into SPCS (sperm cell) carriers, a viability assessment utilizing a Live/Dead assay kit (Calcein AM/ethidium homodimer-1) revealed high levels of cell survival. The analysis revealed an abundance of green fluorescence, indicative of living cells, as shown in Fig. 1e. This result further substantiated the capability of the SPCS carrier system to integrate P6/Uk/PRO NPs and encapsulate DIP without detracting from sperm cells’ viability. Implications of these outcomes for the feasibility and effectiveness of the proposed method in preserving cell vitality throughout the encapsulation process are profound.

An extended analysis was conducted to delve deeper into the comparative viability of sperm cells post-encapsulation across various groups. This involved a detailed examination of fluorescence intensity levels, providing a quantitative measure of live versus dead cells within each sample. The predominance of green fluorescence across the samples not only underscored the minimal cytotoxic effects of the encapsulation process but also highlighted the efficiency of the SPCS carrier in safeguarding cell integrity during and after drug loading. Moreover, the study explored the potential mechanistic aspects that enabled the SPCS carriers to maintain such high levels of cell viability. This included assessing the biocompatibility of the materials used in the formation of the nanoparticles and the carrier system, as well as the physiological impacts of the encapsulation process on sperm cell functions. By comparing these findings with previous studies and control groups that did not undergo nanoparticle encapsulation, significant insights were gained into the innovative approach of using SPCS carriers for drug delivery. The comparative analysis not only validated the superior performance of the SPCS carrier system in terms of preserving cell health but also emphasized its potential application in reproductive biotechnologies and therapeutic delivery systems. This comprehensive investigation into cell viability post-encapsulation not only contributes to the body of knowledge on nanoparticle-mediated drug delivery but also paves the way for future research aimed at optimizing cell-based carriers for various biomedical applications.

### Characterization of DIP-SPCS-P6/Uk/PRO NPs

**Fig. 2:**
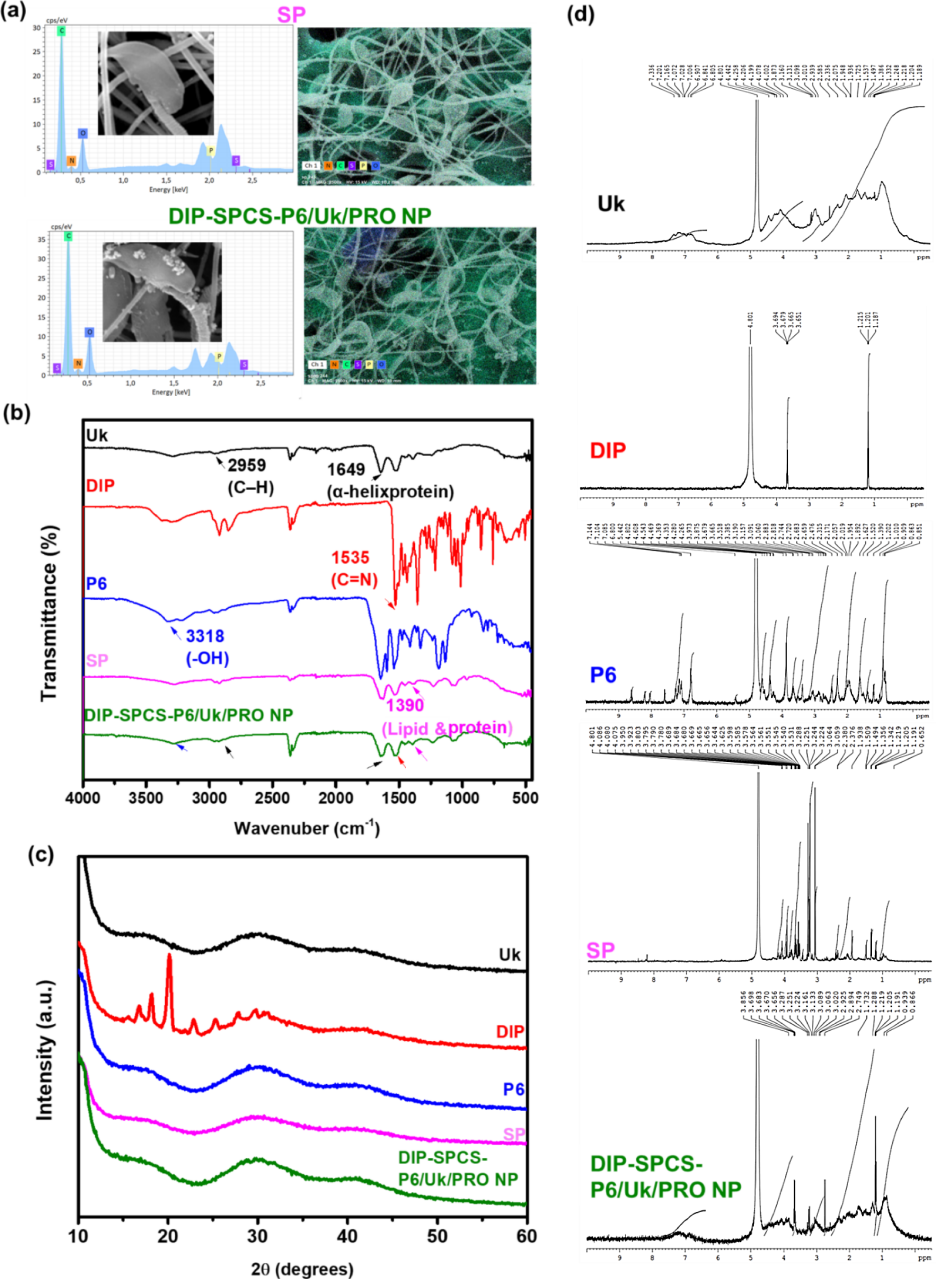
Characterization and related performance test of dipyridamole (DIP)-spermatozoon-propelled cellular submarine (SPCS)-hirudin peptide (P6)/urokinase (Uk)/protamine (PRO) NPs. (**a**) SEM-EDS analysis of DIP-SPCS-P6/Uk/PRO NPs. These data provide a dual-perspective view showcasing on one side, the intricate morphological details of sperm (SP) cells including the head and the flagellum structure. These visual details highlight the structural integrity of SP cells, as evidenced by unbroken plasma membranes and fully intact head membranes and acrosomes. On the other side, the data illustrate the unique bean sprout shape of the SP microcarrier enhanced with DIP-SPCS-P6/Uk/PRO NPs. The rough texture of the DIP-SPCS-P6/Uk/PRO NP microcarrier sharply contrasts with the smooth surface of the standard SP microcarrier, a difference attributed to the integration of PRO-stabilized P6/Uk/PRO NPs. The elemental composition analysis underscored the successful incorporation of DIP, P6, Uk, and PRO into the SP microcarrier, enriching its chemical makeup with higher mass percentages of oxygen (**O**) and carbon (**C**) compared to the SP group alone, signifying the potential of this enhanced microcarrier to be an effective drug-delivery system. (**b**) FTIR spectra of Uk, DIP, P6, SPCSs, and DIP-SPCS-P6/Uk/PRO NPs. FTIR data of DIP-SPCS-P6/Uk/PRO NPs showed the presence of prominent peaks. (**c**) XRD analysis of Uk, DIP, P6, SPCSs, and DIP-SPCS-P6/Uk/PRO NPs. XRD data showed the amorphous or crystalline nature of the DIP-SPCS-P6/Uk/PRO NPs. (**d**) NMR spectra of Uk, DIP, P6, SPCSs, and DIP-SPCS-P6/Uk/PRO NPs. NMR data of Uk, DIP, P6, SP, and DIP-SPCS-P6/Uk/PRO showed prominent peaks and shifts in the end product. NMR spectra showed potentially shifted peaks in the DIP-SPCS-P6/Uk/PRO, indicating the formation of the product

The scanning electron microscopic coupled with energy dispersive x-ray spectroscopic (SEM-EDS) analysis provided pivotal morphological insights, revealing detailed structures and internal linkages within sperm (SP) cells and DIP-SPCS-P6/Uk/PRO NPs, notably between the smooth surface of the sperm head and tail, as illustrated in Fig. 2a. This morphological detail is crucial for a deeper understanding of sperm cell architecture and functionality. The structure of the sperm is visualized, highlighting the acrosomal vesicle and the dense nucleus within the head, as well as the flagellum, which agrees with previously reported findings [45]. A key observation from SEM-EDS data is the prevalence of sperm heads with unbroken plasma membranes, alongside spermatozoa that retain fully intact head membranes and acrosomes, indicating a high degree of structural integrity.

Figure 2a presents SEM-EDS data showcasing the distinctive bean sprout shape of the SP microcarrier, which was enhanced with nanoparticles in the form of DIP-SPCS-P6/Uk/PRO NPs. Notably, the surface of the DIP-SPCS-P6/Uk/PRO NP microcarrier exhibited a rough texture, a stark contrast to the smooth surface observed in the standard SP microcarrier. This textural difference is attributed to the incorporation of PRO-stabilized P6/Uk/PRO nanoparticles, which adds a complex layering to the SP microcarrier. Previous studies acknowledged the effectiveness of SP cells as a potential drug-delivery system [46]. The elemental composition analysis provided by SEM-EDS data revealed that both SP cells and DIP-SPCS-P6/Uk/PRO NP microcarriers contained essential elements such as oxygen (O), nitrogen (N), carbon (C), sulfur (S), and phosphorus (P). A comparative analysis indicated that the DIP-SPCS-P6/Uk/PRO NP group exhibited higher mass percentages of O and C than the SP group alone. This increment suggests the successful integration of DIP, P6, Uk, and PRO components with the SP microcarrier, enhancing its elemental composition.

Delving deeper into the comparison between the two groups, the increased O and C contents in DIP-SPCS-P6/Uk/PRO NP microcarriers could be indicative of the complex molecular structure introduced by the encapsulated drugs and nanoparticles. This structural complexity not only confirmed the successful loading of DIP, P6, Uk, and PRO onto the SP microcarrier but also hinted at the potential for enhanced performance in drug-delivery applications. Moreover, the analysis of the rough interface of the DIP-SPCS-P6/Uk/PRO NP microcarrier compared to the smooth interface of SP cells suggested that the nano-decoration process significantly altered the surface properties of the SP microcarrier. These alterations could potentially improve the microcarrier’s interactions with target cells or tissues, thereby increasing the drug-delivery efficiency. The SEM-EDS image and subsequent elemental analysis underscore the successful development of a nano-enhanced SP microcarrier. The introduction of DIP, P6, Uk, and PRO nanoparticles not only enriched the elemental composition of the SP microcarrier but also modified its surface texture, paving the way for improved drug delivery capabilities. This comparative analysis between the nano-enhanced and standard SP microcarriers highlights advancements in microcarrier technology for more effective therapeutic applications.

Sperm cells, distinguished by their natural shape and proficiency in navigating the female reproductive tract, have emerged as exceptional vectors for deploying treatments against cervical cancer and other gynecological disorders [32]. Sperm cells are known for their inherent ability to swim. This mobility is primarily due to the flagellum, which propels the sperm towards the egg during fertilization [47]. The sperm’s membrane safeguards the encapsulated drugs from dilution by bodily fluids, immune detection, and enzymatic decomposition, thereby efficiently circumventing dose dumping—a significant challenge associated with micellar delivery systems—owing to their robust membrane architecture [32]. This sperm-hybrid micromotor system amalgamates numerous advantageous attributes: it boasts a high capacity for drug loading, autonomous propulsion, the capability for in situ, non-invasive activation for drug release, and enhanced penetration efficiency, thereby ensuring improved drug bioavailability. The introduction of this sperm-hybrid micromotor marks a pioneering advancement in targeted drug delivery, signifying a novel approach that marries biological mechanisms with synthetic engineering to enhance therapeutic outcomes, especially for treating gynecological conditions.

In FTIR analysis revealed the presence of several key functional groups and bonds within the DIP-SPCS-P6/Uk/PRO NPs, providing insights into the structural and chemical fidelity of the encapsulated components (Fig. 2b). Notably, the spectrum identified C-H bonds at 2959 cm^− 1^ [48] in Uk, affirming the successful encapsulation and preservation of this vital component within the formulation. This particular peak is indicative of aliphatic hydrocarbon chains, a foundational structural element in many biological molecules, thereby confirming Uk’s integrity post-encapsulation. Furthermore, the presence of an alpha-helix protein conformation was detected at ca. 1649 cm^− 1^ [49] within the Uk and the nanoparticle formulation. Identifying this specific conformation is critical, as alpha-helix structures are crucial for protein function, suggesting that the proteinaceous components of the formulation maintained their structural integrity after encapsulation.

Additionally, OH bonds, characteristic of P6, were consistently observed at ca. 3318 cm^− 1^, indicating the retention of P6’s chemical composition within the final product. OH groups play a pivotal role in hydrogen bonding and are essential for the solubility and reactivity of many compounds, highlighting the preservation of P6’s functional properties in the nanoparticle assembly. Moreover, the analysis revealed C = N bonds, marked by a peak at approximately 1535 cm^− 1^ in DIP, underscoring the successful integration of DIP into the nanoparticle structure. The presence of C = N bonds is significant as it represents nitrogen-containing compounds that often play critical roles in biological systems, including as components of drugs. In the SP group, a peak at 1390 cm^− 1^ indicated the presence of lipids and cell proteins [50], common in both the SP and DIP-SPCS-P6/Uk/PRO NP formulations. This spectral feature is reflective of the biochemical makeup of the cell, suggesting that the lipid and protein contents crucial for cellular function remained intact within the formulation. The collective spectral data from the FTIR analysis provide compelling evidence of the structural and chemical integrity of the DIP-SPCS-P6/Uk/PRO NPs. By comparing specific absorption peaks with known standards and previous research findings, it is possible to deduce the successful encapsulation and preservation of individual components’ functional structures. This meticulous analysis ensured that the final nanoparticle formulation was structurally sound, retaining the chemical compositions crucial for its intended therapeutic applications. The ability to maintain these structural features post-encapsulation not only demonstrated the effectiveness of the encapsulation process but also reinforced the potential of DIP-SPCS-P6/Uk/PRO NPs as a versatile platform for drug delivery in therapeutic interventions.

The XRD analysis (Fig. 2c) indicated that no clear peaks were seen in the final product, or more precisely DIP-SPCS-P6/Uk/PRO NPs. Because the formulation of Uk, P6, and SPCSs was amorphous and lacked distinctive peaks, the constituent parts likely lacked a well-defined crystalline structure. The idea that the NPs had an amorphous or disordered structure was further supported by the absence of notable peaks in the final product’s XRD pattern. This feature is frequently preferred in drug-delivery systems because it can improve the release and solubility of encapsulated medications, like DIP. It also emphasizes how crucial it is to comprehend the structural characteristics of nanoparticles to judge whether or not they are suitable for therapeutic uses. To summarize, the results of the XRD analysis showed that the DIP-SPCS-P6/Uk/PRO NP formulation did not have a significant crystalline structure. In the NMR analysis (Fig. 2d) of primarily aromatic residues, a combination of signals from various moieties were detected, such as protons linked to esters, anomeric protons in carbohydrates, and double bonds, with the majority of signals coming from carbohydrates. The most obvious sign of protein’s presence was major contributions from protein/peptide structures like amides (N–H), aromatic amino acids (aa), a proton, and methylated side chains (CH_3_). Prominent peaks in Uk, DIP, P6, and SPCSs had slightly shifted in DIP-SPCS-P6/Uk/PRO. This indicated that the products had successfully formed.

**Fig. 3:**
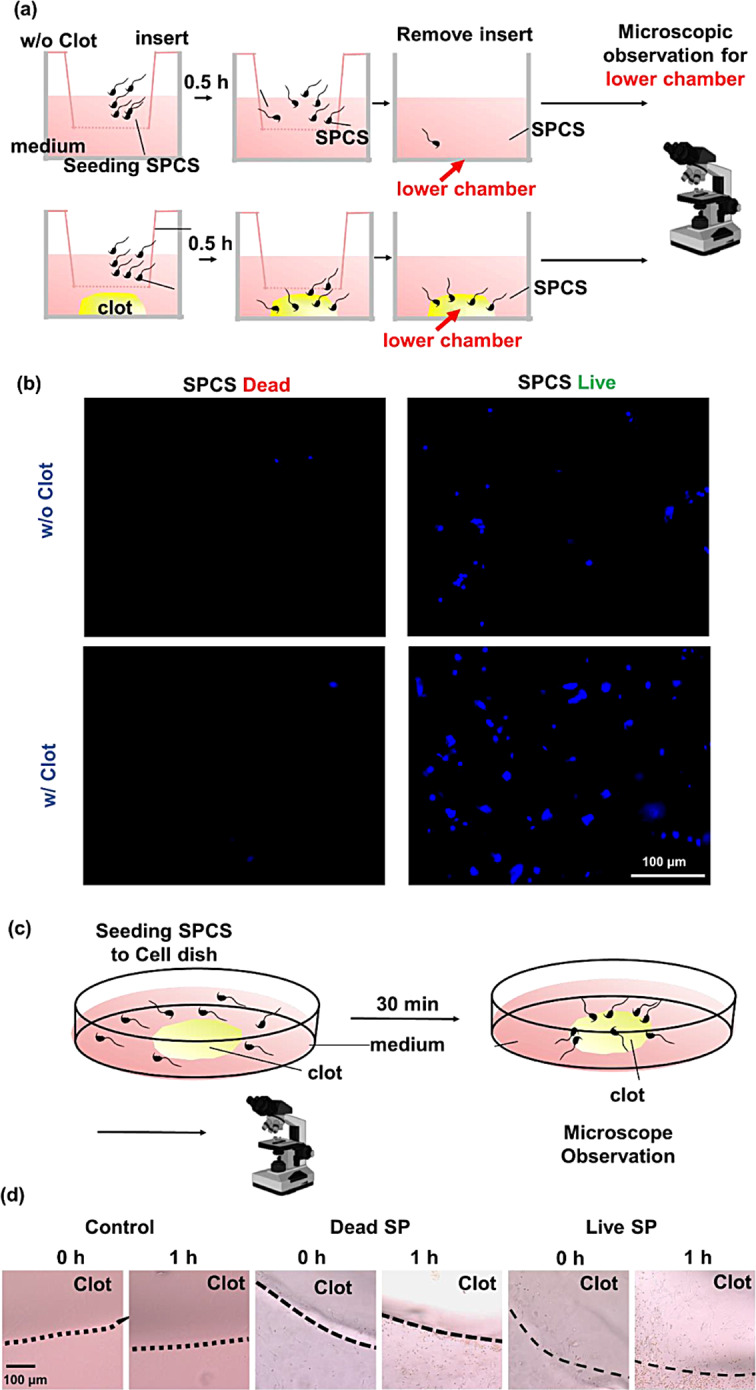
(**a**) Schematic illustration depicting the experiment of spermatozoon-propelled cellular submarine (SPCS) trafficking to a blood clot using a transwell model. Both live and dead SPCSs were used to evaluate their efficiency in interacting with clots. Dead SPCSs were rendered nonviable by treatment with 5% DMSO. (**b**) Microscopic images display the results of the clot-penetration test. Viable SPCSs, stained with DAPI, show a significantly higher penetration into the blood clots compared to non-viable SPCSs and the control group (without clots). The images provide visual confirmation of the superior clot-penetrating ability of viable SPCSs, highlighting their potential for therapeutic applications. (**c**) Schematic illustration depicting the experiment of SPSC trafficking to a blood clot using a 2D model. The 2D model serves to further validate the clot-penetrating capabilities of SPCSs in a different experimental context, complementing the findings from the transwell model. (**d**) Microscopic data from the 2D model corroborate the findings from the model, showing that viable SPCSs have a markedly higher ability to penetrate blood clots compared to non-viable SPCSs.The control group in this experiment, consisting solely of clots without SPCSs, demonstrates the baseline condition for comparison. Microscope drawing was created with BioRender.com

### Clot penetration and in vitro experiments

To study the interaction between SPCSs and clots, both live and nonviable SPCSs were used in in vitro efficiency tests. Nonviable SPCSs were generated by treating them with 5% DMSO. To evaluate the ability of live SPCSs to pierce a clot in the lower chamber and act as a simulated microvascular pore channel, they were seeded on the upper layer of a transwell insert and pre-stained with DAPI.

Both live and dead SPCSs were used in in vitro efficiency tests of SPCS-to-clot interactions (Fig. 3a); the latter were made nonviable by treating them with 5% DMSO. According to the experimental results, live SPCSs (Fig. 3b) showed greater penetration into clots than either the group of dead cells or the control group without clots. The 2D clot-penetration test, as depicted in Fig. 3c, revealed a consistent pattern, confirming earlier findings that viable SPCSs are significantly more effective at infiltrating clots compared to nonviable SPCSs, as shown in Fig. 3d.

**Fig. 4:**
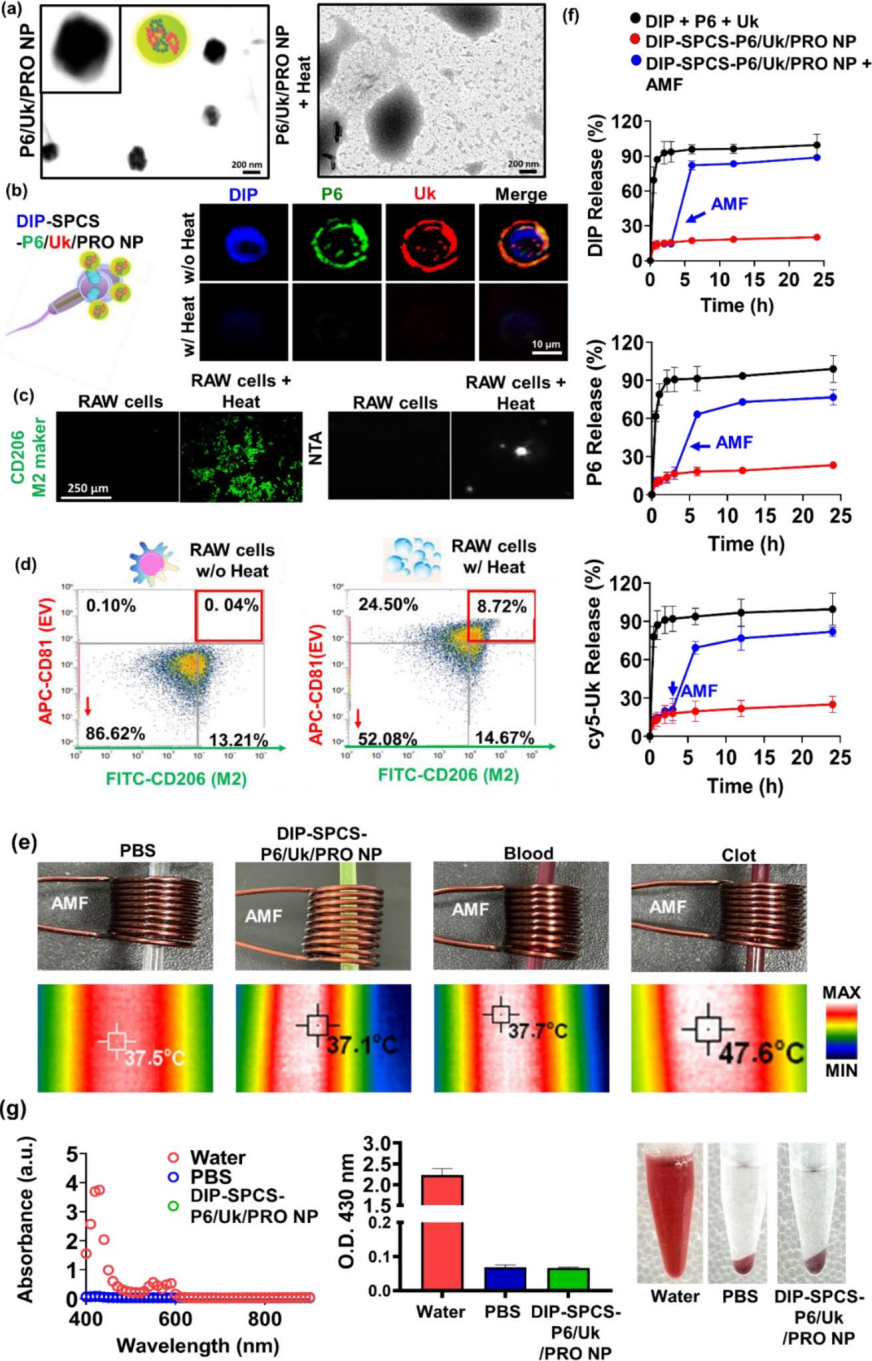
Biological effects of dipyridamole (DIP)-spermatozoon-propelled cellular submarine (SPCS)-hirudin peptide (P6)/urokinase (Uk)/protamine (PRO) NPs. (**a**) TEM and (**b**) fluorescence microscopic data confirmed morphological changes of DIP-SPCS-P6/Uk/PRO NPs following induction of mild hyperthermia. (**c**) Fluorescence microscopic results for M2 (CD206, left) macrophages by a nanoparticle tracking analysis (NTA, right). (**d**) Flow cytometric data demonstrating the production of M2 extracellular vesicles (EVs) by macrophages after mild-hyperthermia treatment. (**e**) Photographic and thermographic data illustrating the mild-hyperthermic effect on blood clots at the physiological temperature (37 °C) under application of an alternating magnetic field (AMF; at 9 V), compared to control groups including PBS and blood without clot formation. (**f**) Drug-release dynamics of DIP-SPCS-CR 110-P6/Cy5-Uk/PRO NPs. These data illustrate the percentages of drugs (DIP, CR 110-P6, and Cy5-Uk) released over time from the formulated nanoparticles in mouse blood solutions. The release profiles were compared between formulations exposed to an AMF for enhanced release and a control group of freely mixed drugs without carrier encapsulation. Enhanced drug release under AMF exposure is also depicted, showing a significant increase in the release rate due to hyperthermia-induced mechanisms. (**g**) Hemocompatibility assessment of DIP-SPCS-P6/Uk/PRO NPs. The data present results of hemolysis assays conducted on rabbit blood to evaluate the hemocompatibility of the formulations. Comparisons were made between the hemolytic ratios observed after 1 h of exposure to nanoparticles, with Milli-Q water as a positive control indicating complete lysis, and a saline solution as a negative control indicating no lysis. The data showcase the formulation’ excellent blood compatibility, with hemolytic ratios well below the 5% threshold defined by ISO/TR 7406 for nonhemolytic materials, indicating the suitability of DIP-SPCS-P6/Uk/PRO NPs for biomedical applications involving blood contact. Experimental results are presented as the mean ± SD

The well-dispersed nanosized morphology of P6/Uk/PRO NPs was revealed by an electron microscopic analysis. But after 20 min of moderate hyperthermia at 45 °C, these nanostructures had transformed into fractured and asymmetrical structures (Fig. 4a). This modification implied that mild hyperthermia was essential for the disintegration of PRO-based polymer nanostructures, which in turn allowed for the regulated release of encapsulated drugs. Furthermore, the presence of blue fluorescence in the fluorescence microscopic results demonstrated additional proof of the intracellular accumulation of DIP in SPCS cells via osmotic processes (Fig. 4b). Additionally, it was observed that P6/Uk/PRO NPs adhered to the extracellular periphery of SPCSs, as indicated by the green fluorescence that came from CR 110, an essential component of P6. The presence and distribution of Uk were confirmed by the red fluorescence that resulted from the conjugation of the amine group of Uk with the Cy5-NHS ester (Fig. 4b). Fluorescence imaging results validated that DIP and P6/Uk/PRO NPs had successfully amalgamated within SPCS cells. Moreover, they demonstrated exceptional mobility and the capacity to pierce deeply into blood clots (Fig. 3). DIP-SPCS-P6/Uk/PRO NPs were the result of the combination of DIP and P6/Uk/PRO NPs. Following mild-hyperthermic treatment, DIP, Cy5-Uk, and CR 110-P6 fluorescence signals decreased, indicating the regulated release of DIP, Uk, and P6 from the SPCSs. The structural and functional characteristics of P6/Uk/PRO NPs, their dynamic interaction with SPCSs, and the controlled release of encapsulated drugs (Fig. 4b) within the cellular environment are all highlighted by these findings.

Based on experimental evidence from fluorescence microscopy, NTA, and flow cytometry (Fig. 4b-d), we noticed that subjecting macrophages to mild hyperthermia, approximately at 45 °C, resulted in a shift towards an anti-inflammatory M2 state characterized by the expression of CD206 (Fig. 4c, left). Moreover, EVs and medications were released by heating these M2 macrophages. The production of submicron-sized particles, or EVs, during hyperthermic treatment of macrophages, was demonstrated by NTA results (Fig. 4c, right). Interestingly, in vitro flow cytometric data showed increased CD206 expression, which is indicative of M2 state macrophages, and CD81, a known marker of macrophage EVs (Fig. 4d). This confirmed that applying mild hyperthermia-induced macrophage polarization into the M2 phenotype, which in turn induced the release of M2-state EVs. It is noteworthy that the anti-inflammatory properties and vascular protective effects of M2-state EVs were widely documented in earlier research studies [51–54].

Thermographic data from this study demonstrated that blood clots with RBCs caused a magnetocaloric effect when exposed to an AMF (of 9 V), which resulted in a significant rise in the clot temperature to a hyperthermic level of 47.6 °C (Fig. 4e). The temperature that the clots reached was significantly higher than the group of DIP-SPCS-CR 110-P6/Cy5-Uk/PRO NPs (37.1 °C) and control group temperatures, which included PBS (37.5 °C) and blood samples (37.7 °C) which did not form clots (Fig. 4e). This astonishing finding suggests that there were significant amounts of RBCs inside the blood clots, which may be the reason for their magnetic responsiveness, high viscosity, and poor heat-dissipation capacity. This method of using a non-invasive magnetocaloric effect for heat therapy has the potential to effectively dissolve or ablate certain blood clots, improving blood vessel patency in the process. Simultaneously, the laden active ingredients that were released during heat treatment showed promise for working in concert with antithrombotic agents. These revelations could improve patient outcomes by reducing the need for invasive interventions while having a significant impact on the development of non-invasive therapeutic modalities for clot-related conditions.

The effective delivery of therapeutic agents from a carrier system is essential for achieving the desired medicinal impacts. Evaluating drug-release dynamics from a given formulation offers critical insights into its potential behavior within a live organism. As depicted in Fig. 4f, the analysis showcased the gradual release rates of drugs (DIP, CR 110-P6, and Cy5-Uk) from the formulated DIP-SPCS-CR 110-P6/Cy5-Uk/PRO NPs in a mouse blood solution, with comparisons made between samples exposed to an AMF and a control group consisting of freely mixed DIP, CR 110-P6, and Cy5-Uk over a defined timeframe.

The initial few hours of the release profile of DIP-SPCS-CR 110-P6/Cy5-Uk/PRO NPs exhibited a biphasic pattern. At first, there was a quick release of approximately 10% of the medications within the first 4 h. This was likely because the pharmaceuticals were spreading out from the outer layers of the DIP-SPCS-CR 110-P6/Cy5-Uk/PRO NPs. Following release stages exhibited a notable decrease in speed, with a moderate and steady discharge of the medication lasting for 24 h. The prolonged-release period, lasting from 4 to 24 h, can be due to the slower diffusion mechanisms, likely occurring through the polymeric carrier core and degradation of the matrix material. This results in the release of around 25% of the medicines by the 24-hour mark. In contrast, the undiluted drug mixture was observed to be fully released within a short period of 2 h, highlighting the effectiveness of the formulations in prolonging the length of drug release.

To examine the effect of AMF-induced hyperthermia on drug release, the DIP-SPCS-CR 110-P6/Cy5-Uk/PRO NPs were subjected to AMF exposure for 5 min. The intervention resulted in a substantial increase in drug release, with the rate of release almost doubling compared to settings without exposure to alternating magnetic fields (AMF). The improved release observed under alternating magnetic field (AMF) conditions can be attributed to the presence of magnetic red blood cells (RBCs) in the solution. When exposed to an AMF, these cells create heat through relaxation. The heat efficiently elevated the temperature of the lipid components of the sperm cell to their phase transition threshold of 42 °C [55], causing a quick release of the included medications. A comparison examination was conducted between the nanoparticle-mediated drug-delivery system and the control setup, revealing that the former exhibits superior capabilities in achieving controlled and sustained release of therapeutic drugs. Moreover, the use of AMF as a stimulus for hyperthermia-induced drug release introduces a new approach to improve the effectiveness and timing of drug administration, particularly in specific therapeutic contexts. This work emphasizes both the advanced capabilities of the DIP-SPCS-CR 110-P6/Cy5-Uk/PRO NPs and their potential to enhance therapeutic results in medical interventions.

An evaluation of the hemocompatibility of formulations is considered critical in the design of biomaterials for in vivo uses [56, 57]. The hemocompatibility of DIP-SPCS-P6/Uk/PRO NPs was evaluated in this study by examining their effect on rabbit blood. The main focus was on measuring hemolysis at a temperature of 37 °C, as shown in Fig. 4g. Milli-Q water was used as a positive control to demonstrate complete lysis, while a saline (PBS) solution served as a negative control to show no lysis. The hemolytic ratios resulting from a 1-hour exposure to DIP-SPCS-P6/Uk/PRO NPs were analyzed, as shown in Fig. 4g. According to the rules of ISO/TR 7406, materials that have hemolytic rates lower than 5% are categorized as nonhemolytic. Therefore, these results suggested that DIP-SPCS-P6/Uk/PRO NPs have a high level of compatibility with blood, highlighting their potential for safe use in biomedical applications that include direct contact with blood.

**Fig. 5:**
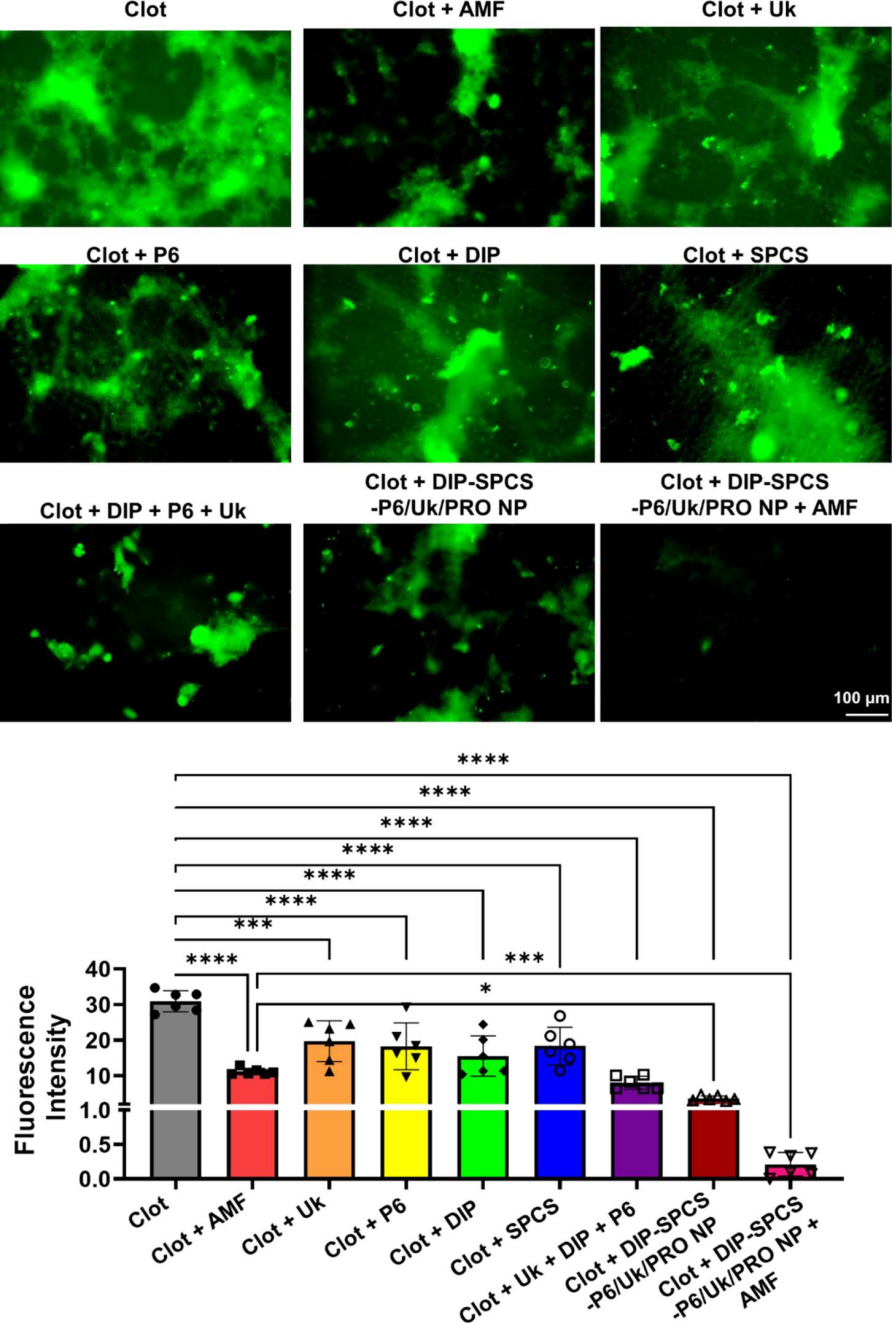
Fluorescence microscopic images of fibrin clots after treatment with urokinase (Uk), hirudin peptide (P6), dipyridamole (DIP), Uk + P6 + DIP, an alternating magnetic field (AMF), SPCS, DIP-SPCS-P6/Uk/PRO NPs, AMF + DIP-SPCS-P6/Uk/PRO NPs. To test DIP-SPCS-P6/Uk/PRO NP’s effectiveness in interacting with blood clots, a sample of FITC-fibrinogen was prepared to enable a fluorescent analysis. Fibrin clots were created in a small Petri dish. A mixture of fibrinogen, red blood cells (RBCs) at a concentration of 10^8^ cells/mL, platelets at a concentration of 10^8^ cells/mL, white blood cells (WBCs) at a concentration of 10^8^ cells/mL, along with 20 µL of CaCl_2_ (0.2 mg/mL), and 2 µL of thrombin (100 U/mL) were subsequently introduced and incubated at 37 ℃. The dishes were then tested with various substances, including DIP-SPCS-P6/Uk/PRO NPs to understand their efficacy in interacting with blood clots. DIP-SPCS-P6/Uk/PRO NPs, when applied with AMF, proved to have highly effective thrombolytic and antithrombotic properties. This was due to their deep penetration, magnetic hyperthermia responsiveness, and synergistic effects of Uk, P6, and DIP. The potential of vascular medical science is truly remarkable. Experimental results are presented as the mean ± SD. The 2-way ANOVA was used to determine statistical significance, as indicated by * *p* < 0.0332, ** *p* < 0.0021, *** *p* < 0.0002, and **** *p* < 0.0001

After observing the drug loading, precise clot penetration, and satisfactory magnetic performances of natural clots under AMF, we proceeded with a FITC-labeled fibrin clot assay to evaluate the structural splitting of the fibrin skeleton. During the in vitro clot-lysis assessment, the loss of green fluorescence from fibrin was monitored for 30 min to determine the degree of fibrinolysis. Results were analyzed, and Fig. 5 depicts representative images of various treatment groups during the 30-minute period. Figure 5 also displays assessment results of “fibrin fluorescence loss” in the imaged clot area during the same period. The use of PBS in the clot application led to no degradation of fibrin, as confirmed through fluorescence microscopy. The fluorescent clot method is exceptionally reliable and represents an advanced technique useful in drug development and diagnostic purposes for examining fibrinolysis and coagulation. During the experiments, clot components such as Uk, P6, DIP, or SPCS being independently included resulted in minimum degradation of fibrin or an antithrombotic effect, as determined by fluorescence microscopy. When combined, Uk, P6, and DIP only produced slight disintegration of fibrin or an antithrombotic effect, also determined by fluorescence microscopy. The use of either an AMF or DIP-SPCS-P6/Uk/PRO NPs resulted in a moderate level of fibrin degradation or an antithrombotic effect. However, the combination of DIP-SPCS-P6/Uk/PRO NP and an AMF produced significant efficacy, compared to groups that received only the clot given Uk, P6, DIP, or Uk + P6 + DIP (refer to Fig. 5). The incredible efficacy of DIP-SPCS-P6/Uk/PRO NPs under AMF application is evident in Fig. 5, where the occlusive clot was almost eliminated. This remarkable achievement in thrombolysis and antithrombotic properties is a result of the deep penetration of DIP-SPCS-P6/Uk/PRO NPs, their magnetic hyperthermia responsiveness, and the synergistic effects of Uk, P6, and DIP. It was inspiring to witness the remarkable potential of vascular medical science.

### In vivo studies of thrombolysis efficacy

**Fig. 6:**
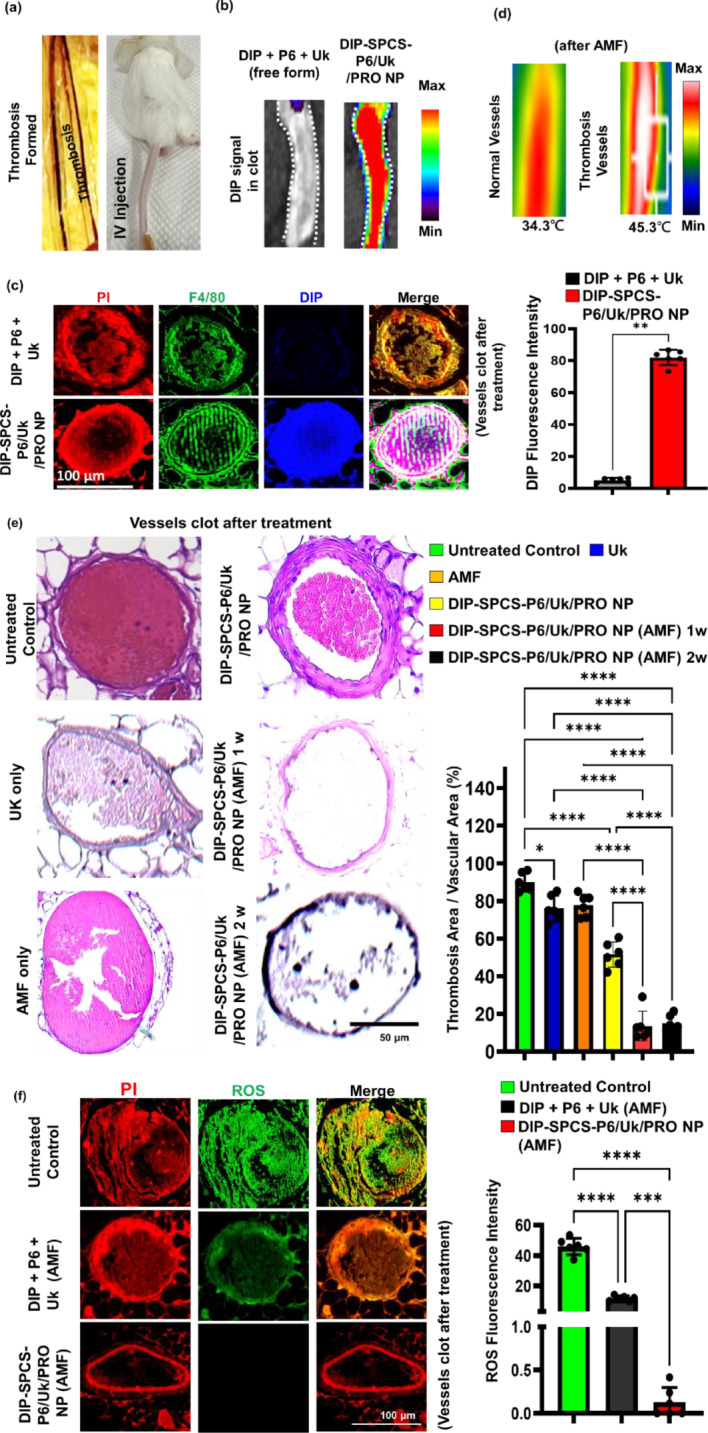
In vivo studies of dipyridamole (DIP)-spermatozoon-propelled cellular submarine (SPCS)-hirudin peptide (P6)/urokinase (Uk)/protamine (PRO) NPs. (**a**) Photographic data illustrating the creation of a mesenteric thrombus mouse animal model using FeCl_3_ and systemic administration via an IV injection. (**b**) Fluorescence IVIS data confirming the targeting and penetration of DIP-SPCS-P6/Uk/PRO NPs into the vascular clot following systemic administration. (**c**) Fluorescence microscopic data demonstrating the accumulation of macrophages within the blood clot and infiltration of administered DIP-SPCS-P6/Uk/PRO NPs into the clot. (**d**) Thermographic data (normal vessel- and thrombus vessel-bearing animals treated with an alternating magnetic field (AMF)), and (**e**) H&E staining data under application of an AMF showing long-term thrombolysis. The experimental groups including thrombus mice + Uk, thrombus mice + AMF, thrombus mice + DIP-SPCS-P6/Uk/PRO NPs, thrombus mice + DIP-SPCS-P6/Uk/PRO NPs (AMF) (1w), and thrombus mice + DIP-SPCS-P6/Uk/PRO NPs (AMF) (2w). (**f**) Fluorescence microscopic data indicating suppression of the inflammatory response in the group treated with DIP-SPCS-P6/Uk/PRO NPs (AMF), compared to animals with untreated thrombi and those that received free-form drugs (DIP + P6 + Uk) plus AMF. Experimental results are presented as the mean ± SD. Quantitative analysis of microscopic images was performed using ImageJ software. The mean ± standard deviation (SD) of experimental results is presented. The 2-way ANOVA was used to determine statistical significance, as indicated by * *p* < 0.0332, ** *p* < 0.0021, *** *p* < 0.0002, and **** *p* < 0.0001

The ferric chloride filter paper was used to induce thrombi in this animal model of mesenteric vascular thrombosis, as shown in Fig. 6a. Following systemic administration, IVIS imaging (Fig. 6b) and a tissue fluorescence section analysis (Fig. 6c) were used to obtain in vivo fluorescent data indicating the drug distribution within thrombi. These observations demonstrated the in vivo DIP-SPCS-P6/Uk/PRO NP mobility and targeting ability, as well as their accumulation and penetration within the thrombotic region. Interestingly, the DIP-attributed fluorescence signal functioned as a representative drug presence marker (Fig. 6b, c). As demonstrated by the green fluorescence of F4/80 (Fig. 6c), the thrombus microenvironment was characterized by infiltration of macrophages, which is consistent with previous research [58] and our current findings. All of these results provide strong evidence that the system can deliver and accumulate DIP-SPCS-P6/Uk/PRO NPs specifically within thrombus sites in vivo. Macrophage infiltration more strongly emphasized their possible function in thrombus microenvironments. These data revealed the drug-delivery system’s therapeutic potential, especially concerning its ability to accurately target and treat thrombotic regions.

DIP-SPCS-P6/Uk/PRO NPs were administered to thrombus-bearing animals and exposed to an alternating magnetic field (AMF) of 9 V via a ca. 4.5-cm coil, examining both scenarios where the vessel or the thrombus-bearing mouse was within the coil. Thermographic studies, as depicted in thermal image Fig. 6d and photographic as well as temperature data Fig. S2a-b, were conducted under the servereal conditions using a thermal imaging/record device. The results showed that normal blood vessels did not exhibit a significant magnetocaloric effect, failing to reach hyperthermic temperatures when exposed to AMF. However, thrombus-affected vessels displayed a different response; the concentrated lesion, increased blood viscosity, and the accumulation of magnetic RBCs substantially hindered heat dissipation. This led to a pronounced magnetocaloric effect, with temperatures rising to hyperthermic levels of about 45 °C in both scenarios. This selective heating in thrombus-affected areas highlights the potential of DIP-SPCS-P6/Uk/PRO NPs combined with AMF in targeted hyperthermic treatment, promising a focused approach to thrombolysis without affecting surrounding healthy tissues.

Following a treatment period ranging from 1 to 2 weeks, subjects were euthanized for further examination. A detailed histopathological evaluation was conducted on thrombus lumen sections, which were stained with hematoxylin and eosin (H&E). The remaining clots of thrombus mice + Uk (ca. 75%), thrombus mice + DIP-SPCS-P6/Uk/PRO NPs (ca. 52%), and thrombus mice + AMF (ca. 76%). The underlying mechanisms through which magnetic hyperthermia and spermatozoon propulsion synergistically enhance thrombolysis and ischemia mitigation have been elucidated. According to the microscopically histological data, the thrombolysis efficacy of the thrombus mice + AMF (magnetic hyperthermia alone) or the spermatozoon propulsion (DIP-SPCS-P6/Uk/PRO NPs) alone is insufficient (Fig. 6e).

This analysis revealed a significant outcome of the combined therapy involving AMF treatment and the targeted delivery of DIP-SPCS-P6/Uk/PRO NPs. The efficacy of this treatment was evident by dissolution of thrombi, with approximately 85% of the clot material resolved, leaving behind less than 15% of the original clot volume (Fig. 6e). This outcome not only highlights the effectiveness of the treatment in clot dissolution but also indicates its potential for the long-term prevention of thrombus recurrence. To extend the discussion and offer a more in-depth comparative analysis, results from this combined treatment approach were contrasted with outcomes observed in an untreated control group and those treated with Uk. The quantitative data underscored a stark difference in the efficiency of thrombus resolution among the groups. While the untreated control group and those receiving Uk treatments showed a lesser degree of clot dissolution, the combination therapy group demonstrated a markedly higher success rate, substantiating the synergistic effect of AMF treatment coupled with DIP-SPCS-P6/Uk/PRO NPs. Previous findings provided valuable insights into the mechanisms behind nanoformulation interactions with thrombotic tissues and the role of AMF in enhancing therapeutic outcomes [59, 60].

Additionally, the untreated thrombus group showed the strongest presence of reactive oxygen species (ROS) as demonstrated by bright DCF green fluorescence (Fig. 6f), indicating a high level of vascular inflammation within the thrombus. This was revealed by a fluorescence analysis of thrombus sections. However, a partial decrease in vascular inflammation within the thrombus was noted when animals with a thrombus were treated with the AMF magnetocaloric effect using freely administered drug components (DIP + P6 + Uk). Importantly, thrombus vessel inflammation was significantly reduced by DIP-SPCS-P6/Uk/PRO NP treatment via AMF-induced hyperthermia. These compelling results provided strong evidence for the efficacy of the combined therapeutic approach in thrombus resolution and thrombus recurrence mitigation. This approach included DIP-SPCS-P6/Uk/PRO NPs and AMF-induced hyperthermia. Furthermore, outcomes highlighted the strategy’s therapeutic potential in reducing vascular inflammation within thrombi, which together open new avenues for creating innovative approaches to thrombus management and avoiding recurrent thrombotic events.

**Fig. 7:**
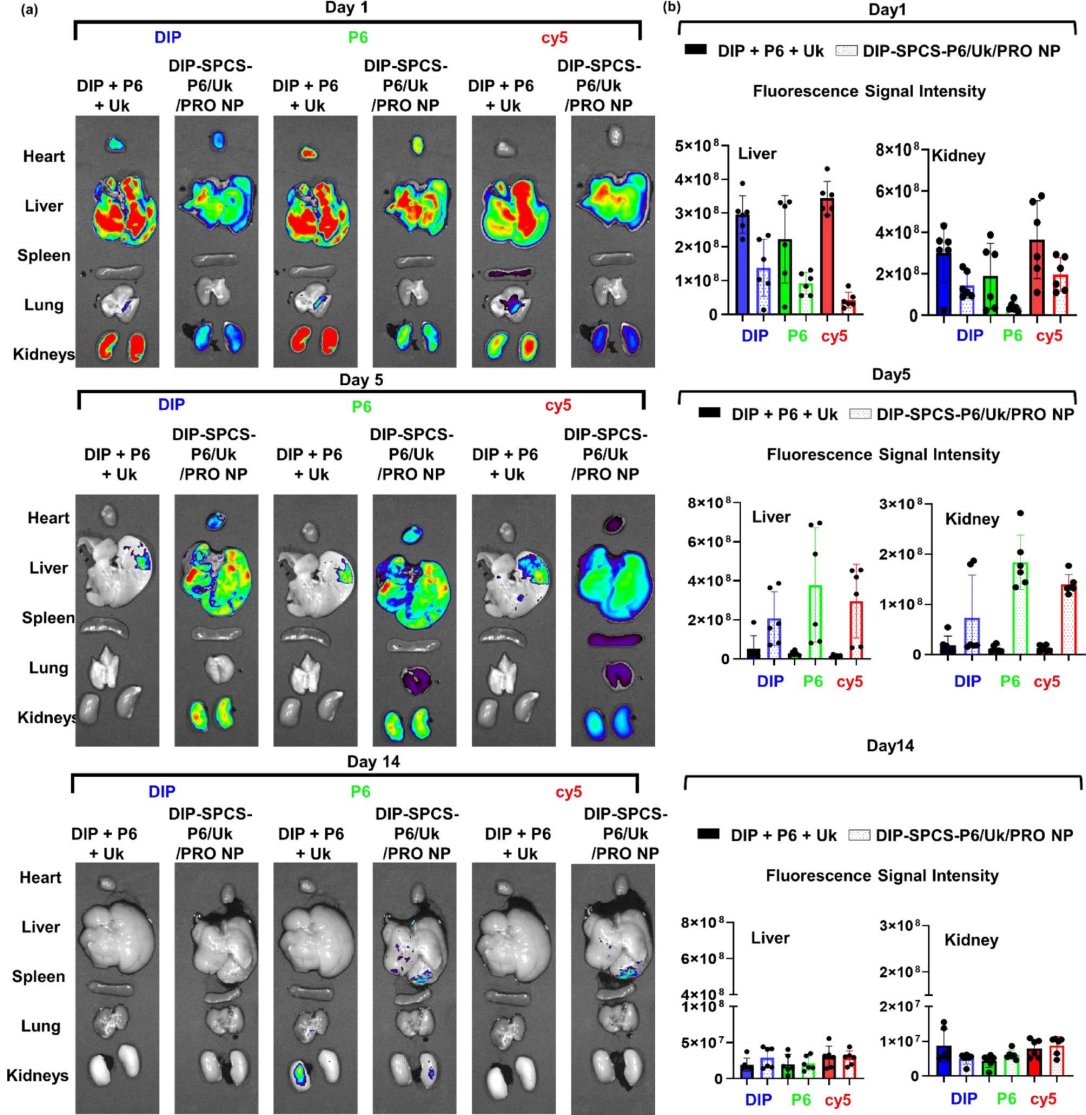
IVIS biodistribution investigations. (**a**) Qualitative and (**b**) quantitative thrombus IVIS data from thrombus-bearing mice that received free-form drugs (CR 110-hirudin peptide (P6) + dipyridamole (DIP) + Cy5-urokinase (Uk)) and thrombus-bearing mice that received DIP-spermatozoon-propelled cellular submarine (SPCS)-CR 110-P6/Cy5-Uk/protamine (PRO) NP). The mean ± standard deviation (SD) of experimental results is presented

The investigation included detailed assessments of the biodistribution and pharmacokinetic profiles of two distinct formulations: the free drug (CR 110-P6 + DIP + Cy5-Uk) and the formulated-based group (DIP-SPCS-CR 110-P6/Cy5-Uk/PRO NPs), fluorescently tagged for visualization. This examination was essential to understand how these formulations were distributed throughout the body under physiological conditions typical of thrombus-bearing mice. Utilizing optical fluorescence imaging technology (IVIS, as illustrated in Fig. 7a and b), we tracked the presence of three pharmacologically active agents (CR 110-P6, DIP, and Cy5-Uk, which are fluorescently labeled) across the two formulations in vivo.

Our IVIS analysis revealed a marked disparity in the pharmacokinetic behaviors of the free-form drugs compared to their formulated counterparts. Specifically, the free-form drugs exhibited rapid declines in detectable levels across various tissues, with significant reductions observed at intervals of 5 and 14 days post-administration. In stark contrast, the formulated DIP-SPCS-CR 110-P6/Cy5-Uk/PRO NP group demonstrated a notably slower rate of signal decay in soft tissues (including the heart, liver, spleen, lungs, and kidneys), indicative of a prolonged systemic presence.

**Fig. 8:**
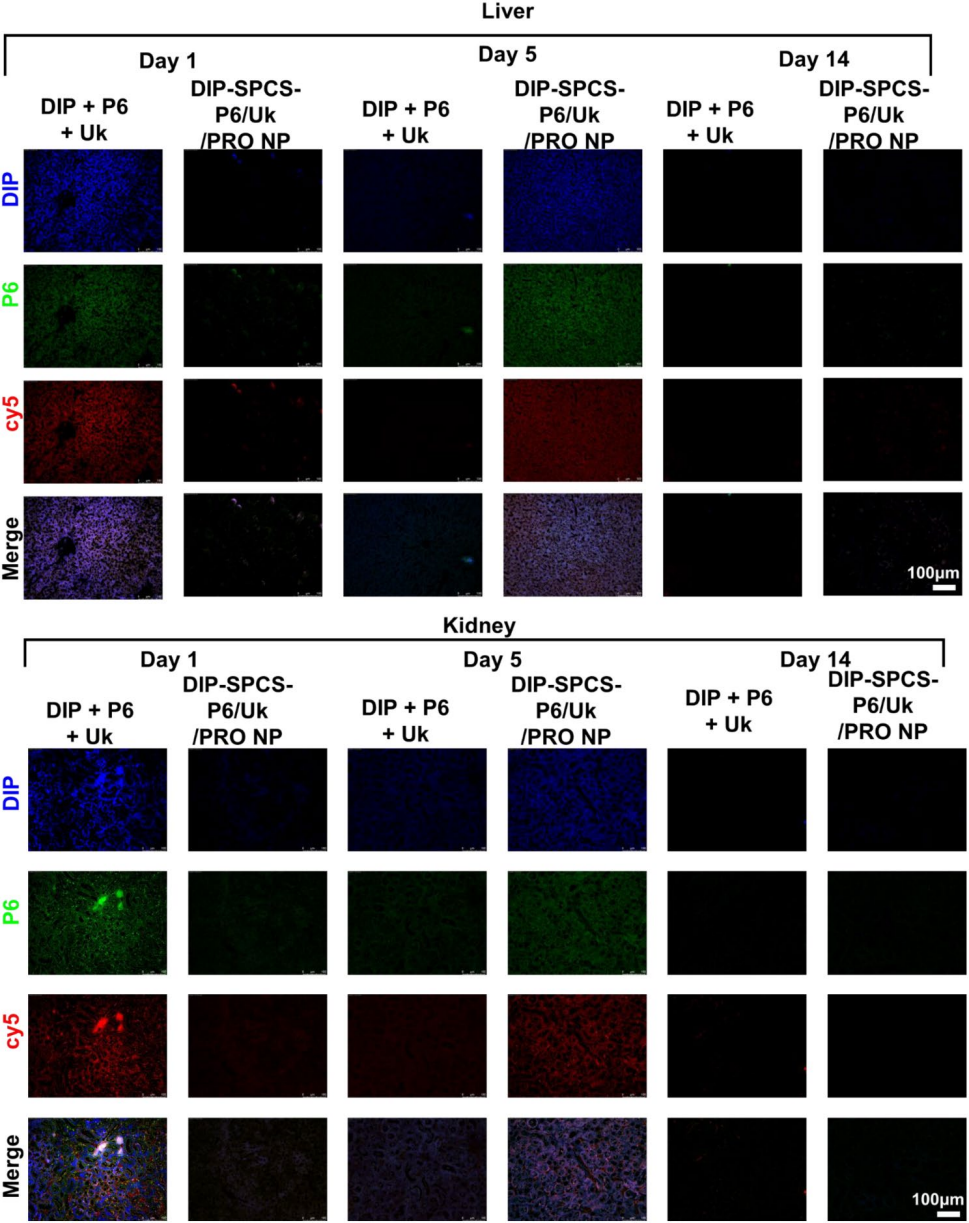
In vivo fluorescence microscopic data of thrombus-bearing mice that received free-form drugs (CR 110-hirudin peptide (P6) + dipyridamole (DIP) + Cy5-urokinase (Uk)) and thrombus-bearing mice that received DIP-spermatozoon-propelled cellular submarine (SPCS)-CR 110-P6/Cy5-Uk/protamine (PRO) NPs

To corroborate these findings, colocalization studies integrating IVIS with fluorescent microscopy (Fig. 8) were employed, affirming the enduring stability of the DIP-SPCS-CR 110-P6/Cy5-Uk/PRO NP complex. Ex vivo assessments provided further insights, showing detectable levels of both formulations across all evaluated organs shortly after administration. However, by the fifth day, fluorescent signals of the free drugs had markedly diminished, in stark contrast to the enduring presence of those of the DIP-SPCS-CR 110-P6/Cy5-Uk/PRO NP group, which were especially pronounced in the kidneys and liver. This observation underscores the enhanced pharmacokinetic profile offered by the stealth capabilities of the SPCS-formulated system, ensuring extended retention of therapeutic agents within the body. By the 14th day, the presence of both formulations in tissues had largely dissipated, indicating a convergence in the ultimate fate of the drugs, characterized by their eventual elimination and degradation. These findings not only highlight the superior therapeutic efficacy and controlled release properties of the DIP-SPCS-CR 110-P6/Cy5-Uk/PRO NP formulation but also underscore its biosafety and biodegradable attributes. This comprehensive analysis sheds light on the differential biodistribution and pharmacokinetics of free-form drugs versus nanoparticle-encapsulated drugs, emphasizing the potential of SPCS-formulated system technology to enhance drug delivery and efficacy in thrombus treatment strategies. The extended discussion and detailed comparison between the groups, enriched with quantitative data, further contribute to our understanding of these complex pharmacological interactions.

### Assessment of in vivo biological vascular healing, functional recovery, and ischemia prevention

**Fig. 9:**
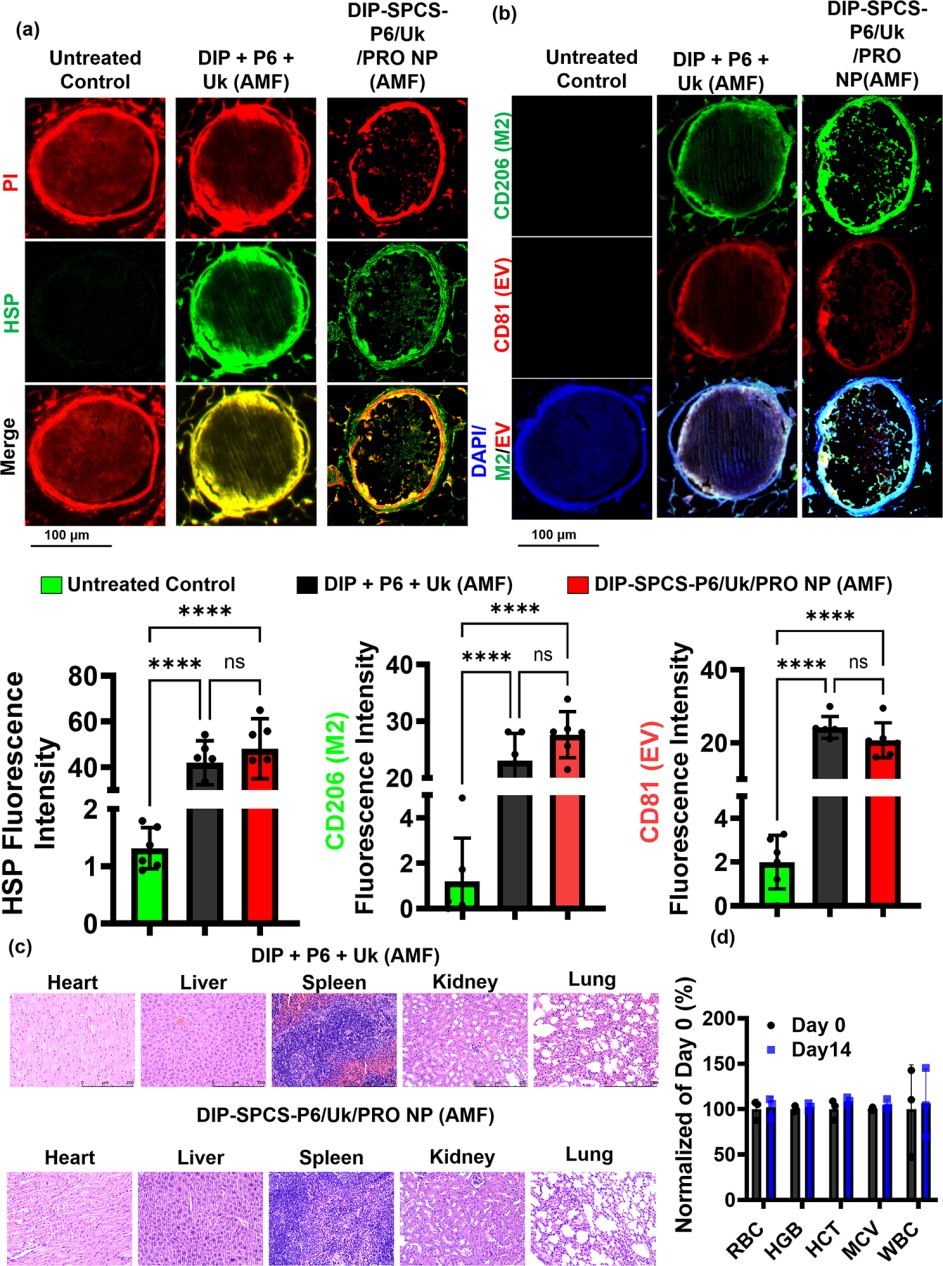
In vivo protective effect of dipyridamole (DIP)-spermatozoon-propelled cellular submarine (SPCS)-hirudin peptide (P6)/urokinase (Uk)/protamine (PRO) NPs. (**a**) Microscopic data demonstrating the induction of vascular heat shock protein 70 (HSP70) in thrombus-bearing animals treated with DIP-SPCS-P6/Uk/PRO NPs plus alternating magnetic field (AMF) application compared to untreated thrombus-bearing animals and similar as thrombus-bearing animals that received free-form drugs (DIP + P6 + Uk) plus an AMF. The experimental groups including thrombus mice, thrombus mice + DIP + P6 + Uk + AMF, thrombus mice + DIP-SPCS-P6/Uk/PRO NPs + AMF. (**b**) Microscopic data illustrating the presence of M2 macrophage-derived extracellular vesicles (EVs) in the vasculature of thrombus-bearing animals treated with DIP-SPCS-P6/Uk/PRO NPs plus AMF application compared to untreated thrombus-bearing animals and similar as thrombus-bearing animals that received free-form drugs (DIP + P6 + Uk) plus an AMF. (**c**) Microscopic data from soft-tissue H&E staining confirming the biosafety and biocompatibility of DIP-SPCS-P6/Uk/PRO NPs in thrombus-bearing animals. (**d**) Comparative blood biochemical analysis over 14 days. The data display results of a comprehensive blood biochemical analysis comparing key hematological parameters between the control group (untreated) on day 0 and after completion of a 14-day treatment period. The parameters analyzed include red blood cell (RBC) count, hemoglobin (HGB) levels, hematocrit (HCT), mean corpuscular volume (MCV), and white blood cell (WBC) count. The data presented aimed to assess the potential hematological impacts of the treatment, with a focus on ensuring the absence of adverse effects. Each bar or data point represents an average value obtained from the subject group, with error bars indicating the standard deviation (SD) to reflect the variability within the group. The stability across these parameters suggests the treatment’s biocompatibility and its nontoxic nature regarding hematological health. The data highlight the protective effect of DIP-SPCS-P6/Uk/PRO NPs in thrombus-bearing animals. The mean ± standard deviation (SD) of experimental results is presented. The 2-way ANOVA was used to determine statistical significance, as indicated by * *p* < 0.0332, ** *p* < 0.0021, *** *p* < 0.0002, and **** *p* < 0.0001

A fluorescence immunostaining analysis of sections was performed after thrombus treatment to assess expressions of EVs, or CD81 (an EV marker), a heat shock protein (HSP70), and CD206 (an M2 marker). Findings showed that the administration of free-form drugs (DIP + P6 + Uk), DIP-SPCS-P6/Uk/PRO NPs in thrombus-bearing animals, in combination with the magnetocaloric effect induced by an AMF, resulted in the production of HSP70 (green fluorescence, Fig. 9a) and the distribution of M2-state EVs (green (M2) and red (EV) fluorescence, Fig. 9b) in vessel walls. The thrombus-untreated group, on the other hand, exhibited no notable HSP70 production or M2-state EV distribution. These results implied that the formation of protective HSP70 in vessel walls was initiated by the magnetocaloric effect (AMF), which raised the temperature to mild hyperthermia, and that M2-state EVs were released by infiltrating macrophages. In order to aid in blood vessel repair, reduce vascular inflammation, prevent relapse, and shield blood vessels from further damage, this system made it easier for blood vessel walls to absorb the released active ingredients. This naturally driven AMF, in turn, promoted the production of M2-state EVs and HSP70. In a final analysis, this strategy provided accurate medication administration for thrombus treatment and prevention. Furthermore, following treatment with the AMF magnetocaloric effect, results of the soft-tissue H&E section analysis (Fig. 9c) showed the good biocompatibility of the DIP-SPCS-P6/Uk/PRO NP carrier system in organ tissues. These encouraging results lend credence to the possibility of additional clinical research and implementation of this carrier system.

Data derived from blood biochemical analyses, as illustrated in Fig. 9d, indicate the absence of negative impacts on various hematological parameters. This comparison spans from the baseline (day 0, before any treatment) to 14 days post-treatment, covering both the untreated control group and those that received treatment. Specifically, the study focused on key indicators such as RBC counts, HGB levels, HCT, MCV, and WBC counts. Initial observations revealed stable levels of RBC, HGB, HCT, MCV, and WBC between the pretreatment phase and after completion of the 14-day treatment regimen. This consistency suggests that the therapeutic intervention did not adversely affect these critical blood components, indicating a high degree of biocompatibility and safety of the treatment.

**Fig. 10:**
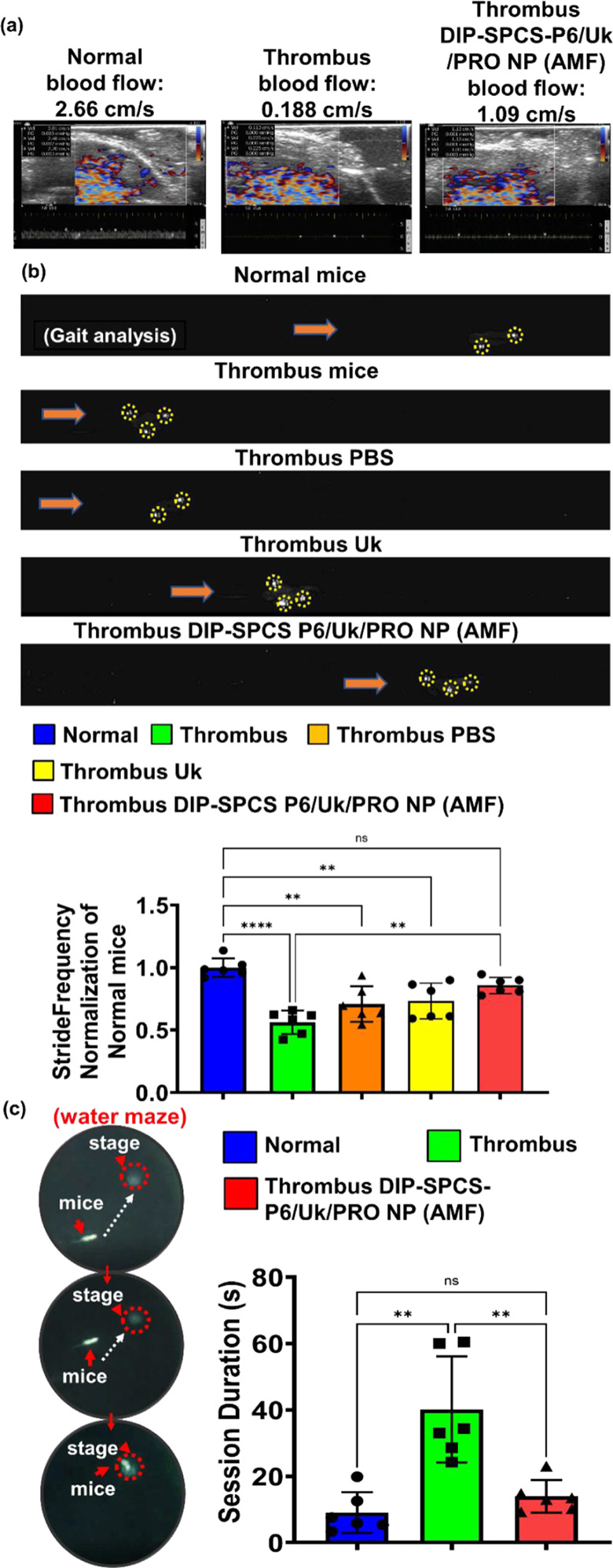
Recovery of vascular blood flow and functional performance in untreated thrombus-bearing mice, thrombus-bearing mice treated with dipyridamole (DIP)-spermatozoon-propelled cellular submarine (SPCS)-hirudin peptide (P6)/urokinase (Uk)/protamine (PRO) NPs plus alternating magnetic field (AMF) application, and normal mice. (**a**) Ultrasound assessment. (**b**) Gait assessment. The experimental groups included normal mice, thrombus mice, thrombus mice + PBS, thrombus mice + Uk, and thrombus mice + DIP-SPCS-P6/Uk/PRO NPs + AMF. (**c**) Water maze assessment. The mean ± standard deviation (SD) of experimental results is presented. The 2-way ANOVA was used to determine statistical significance, as indicated by * *p* < 0.0332, ** *p* < 0.0021, *** *p* < 0.0002, and **** *p* < 0.0001

The integration of US assessments (Fig. 10a) and comprehensive behavioral analyses (Fig. 10b, c) illuminates the therapeutic efficacy of DIP-SPCS-P6/Uk/PRO NPs when used in conjunction with AMF therapy in thrombus-affected animal models. Initially, US evaluations revealed a marked enhancement in vascular blood flow among subjects treated with DIP-SPCS-P6/Uk/PRO NPs and AMF, where the measured blood flow rate was 1.09 cm/s. This rate, while an improvement, was lower than the healthy baseline of 2.66 cm/s but demonstrated a considerable advancement towards normal vascular function compared to the untreated, thrombus-afflicted counterparts. This improvement underscores the potent impact of the treatment on vascular perfusion and the restoration of blood flow within affected vessels.

In the walkability assessment (Fig. 10b), the thrombus mice group exhibited the lowest stride frequency. Minor improvements in stride frequency were observed in the thrombus mice treated with PBS and thrombus mice treated with Uk when compared to the untreated thrombus mice group. However, the thrombus mice treated with DIP-SPCS-P6/Uk/PRO NPs and exposed to an AMF demonstrated a enhancement in stride frequency, surpassing that of the thrombus mice, thrombus mice + PBS, and thrombus mice + Uk groups. The performance of the thrombus mice + DIP-SPCS-P6/Uk/PRO NPs + AMF group closely mirrored the stride frequency observed in the healthy control group, indicating a substantial restoration of normal walkability.

Furthering this examination, behavioral assessments conducted on treated animals showcased significant improvements in mobility, specifically noted in their walking and swimming capabilities. These behavioral improvements, as depicted in Fig. 10b and c, approached levels observed in healthy animals, thereby indicating a substantial recovery in functional abilities post-treatment. Such enhancements in both vascular and behavioral parameters affirm the DIP-SPCS-P6/Uk/PRO NPs, coupled with AMF therapy, as a formidable therapeutic strategy in managing and healing vascular impairments in thrombus-bearing animals.

Delving deeper into the implications of these findings, it became indication that the synergistic application of DIP-SPCS-P6/Uk/PRO NPs and AMF therapy not only facilitated vascular recovery but also contributed to the significant restoration of normal animal behaviors impacted by thrombosis. This dual approach of potentially addressing both physical obstructions in blood flow and subsequent behavioral ramifications establishes a comprehensive therapeutic paradigm, promising advancement in thrombus management. Moreover, the observed outcomes offer a glimpse into potential translational applications of this therapeutic strategy, to enhance therapeutic outcomes and quality of life. The ability of this innovative treatment to restore vascular functionality and improve overall mobility holds immense promise in the context of human thrombus management, paving the way for future clinical research and potential therapeutic applications. The combined use of DIP-SPCS-P6/Uk/PRO NPs and an AMF presents a novel and effective method for promoting vascular healing and functional restoration in thrombus-bearing models. The favorable outcomes from the US and behavioral evaluations underscore the potential of this approach in advancing thrombus treatment protocols and improving patient care.

## Discussion

Many obstacles remain in the way of developing conventional thrombolytic and antithrombotic medications. US technology, thermal therapy (including the photothermal effect), and mechanical therapy have emerged as popular methods for treating thromboses. But as they go deeper into skin tissue, the energy of both near-infrared (NIR) and US light sources significantly decreases. The maximum depth of US penetration into tissues is approximately 5 cm, whereas NIR light penetration can only reach less than 1 cm. These drawbacks make it difficult to use these techniques clinically, particularly when treating deeper vascular lesions [61–63].

On the other hand, clinical uses of magnetic hyperthermic therapy include the treatment of localized tumors [64]. To produce a therapeutic effect, this method makes use of magnetic nanoparticles, such as Fe_3_O_4_, which can produce heat when exposed to an AMF. Compared to photothermal therapy, magnetothermal therapy has several advantages, such as selective tumor targeting and deeper tissue penetration. It has consequently grown to be an important area of study in the field of nanomedicine, especially in oncology. To fill a critical gap in the field, some research groups have begun investigating the use of magnetothermal techniques to dissolve blood clots in the last 1 to 2 years [60].

These investigations frequently use magnetic nanoparticles, especially Fe_3_O_4_, which may raise questions about their biocompatibility in the context of thrombus lesions. Because of the abundance of H_2_O_2_, materials of this kind can cause the Fenton reaction in the thrombus area. Extreme oxidative stress and toxicity may arise from this reaction’s production of extremely reactive hydroxyl radicals Reactive species can have negative effects on normal cells and tissues, including the nervous system. As a result, problems like impaired kidney function and other serious problems may occur. Thus, after thrombus treatment, it is imperative to address the efficient removal of such materials from the body. Nevertheless, corresponding research addressing this crucial factor has been lacking up to this point [65, 66].

A significant portion of magnetic ferrous iron substances in the body is stored within RBCs. In the context of thrombus formation, substantial accumulation of RBCs occurs within blood vessels affected by the clotting process [67]. This pathological characteristic gives rise to the development of a magnetocaloric effect within the affected vessel, as depicted in Fig. 6d. Generation of this magnetocaloric effect subsequently triggers a cascade of therapeutic outcomes, as illustrated in Figs. 6, 7 and 8. Importantly, this innovative technology eliminates the need to add toxic substances. Instead, it relies on the inherent accumulation of a large quantity of magnetic RBCs specifically within the thrombus lesion site, thus enabling the production of a magnetocaloric effect by applying an AMF. This method successfully avoids problems caused by tissue depth-related rapid energy decay, making it a viable option for additional research and possible clinical use [68].

Sperm cells have extraordinary motility, similar to that of bacterial flagella, which allows them to move in a viscosity-directed manner and swim in watery environments. They have traits like being able to pass through physical barriers, they have a ca. 4-day lifespan, and they can transport hydrophilic medications. For treating thrombi, the SPCS carrier system offers a novel, useful, and important drug-delivery method. Even though bacteria and other motile cells have been the subject of much research as drug carriers, questions about their biocompatibility continue to be raised. Few studies have looked into their use in anticancer research or how they combine with magnetic materials to improve blood flow in vitro models. Our approach, on the other hand, used biocompatible SPCSs as drug carriers, taking advantage of their viability and motility to efficiently enter blood clots and administer medications [3].

SPCSs’ viscosity-driven behavior causes them to accumulate inside clots like sperm binding and fusing with an egg [32]. Therefore, it is anticipated that SPCS carriers will gather at the site of thrombus formation. These motile cells, once loaded with antithrombotic and fibrinolytic drugs, pierce the thrombus and release the drugs from their submarine carriers when exposed to an AMF that causes magnetic hyperthermia. Through stimulating infiltrating macrophages within a thrombus, this process causes lesion vessels to vasodilate and produces M2 anti-inflammatory EVs. As a result, HSP expression is induced in the vessel wall, and M2 EVs and delivered therapeutics (Uk, DIP, and P6) are effectively absorbed. This series of actions guarantees precise and targeted drug delivery to the thrombus, thereby reducing the risk of neural damage, preventing thrombus recurrence, and producing an effective antithrombotic effect. Significantly, not much has been studied about macrophage infiltration into thrombus microenvironments, their capacity to produce M2 EVs through intra-thrombus magnetic hyperthermia, or their function in controlling HSP expression to reduce vascular inflammation, shield endothelial cells, and avert brain damage.

Our suggested technology provides a quick, practical, and simple preparation method while minimizing chemical modifications and unfavorable biological reactions. This paper introduces an innovative methodology to tackle complex issues associated with thrombotic diseases, which represent a significant and widespread public health concern on a global scale. This study presents the development of microcellular submarines (SPCSs) propelled by spermatozoa, which were designed to carry anticoagulant medications. Additionally, these submarines were modified through the incorporation of formulation. The aforementioned superparamagnetic colloidal system (SPCSs) exhibited remarkable capabilities in targeting blood clots, facilitating drug release via magnetic hyperthermia, and exerting anti-inflammatory properties.

In the context of an in vivo mouse model, this particular system demonstrated a noteworthy thrombolytic efficiency of ca. 80%, thereby mitigating the potential risk of ischemic injury. The novel methodology possesses the capacity to revolutionize the management of thrombotic diseases and presents a highly efficient framework for the targeted and regulated administration of pharmaceutical agents. The results of this study offer significant potential for enhancing thrombus treatment and overall healthcare outcomes on a global scale.

The transition from murine models to human application presents several challenges that necessitate careful consideration to ensure successful clinical translation of the innovative SPCSs designed for targeted thrombolytic therapy. We outline these challenges, and potential strategies for overcoming them, and delve into scalability and biocompatibility concerns. The process of scaling up the production of SPCSs for clinical application involves several hurdles, such as ensuring consistent quality and functionality across batches and developing protocols for large-scale manufacturing that comply with regulatory standards. A potential strategy to address these challenges involves the adoption of automated bioreactor systems that can control and monitor the conditions necessary for the consistent production of SPCSs. Additionally, rigorous quality control measures and validation processes must be established to ensure that each batch meets necessary therapeutic and safety standards.

While SPCSs demonstrated promising results in murine models, the biocompatibility of these systems in humans must be thoroughly evaluated. This includes assessing the potential immunogenicity of the SPCSs and the formulation used for drug loading and release. Strategies to mitigate biocompatibility concerns include using human-compatible materials for the construction of the SPCSs and conducting comprehensive in vitro and in vivo studies to assess immune responses. Furthermore, encapsulation techniques can be optimized to minimize exposure of the body to the raw materials of the nanoparticles, thereby reducing the likelihood of adverse immune reactions. Optimizing the drug-loading capacity and release kinetics for human applications is crucial. The drug release must be controllable and precise, effectively targeting the thrombus without affecting surrounding tissues. Advanced nanotechnology techniques, such as stimulus-responsive materials that respond to changes in the thrombus microenvironment or external magnetic fields, could offer solutions for the controlled release of therapeutics.

Recent studies explored the use of magnetic nanomaterials for hyperthermia-induced thrombolysis, demonstrating the potential of this approach for dissolving blood clots without invasive procedures [69]. Assessing the biocompatibility of nanocarriers is critical for their safe use in humans [70]. The use of sperm cells as drug carriers has been investigated for targeted therapy, leveraging their natural motility and ability to navigate through viscous environments [71]. The clinical translation of SPCSs for targeted thrombolytic therapy presents several challenges, including scalability, biocompatibility, and optimization of drug-loading and -release mechanisms. Addressing these challenges requires a multidisciplinary approach, leveraging advances in nanotechnology, biomedicine, and materials science. By overcoming these hurdles, SPCSs have the potential to revolutionize the treatment of thrombotic diseases, offering a minimally invasive, highly targeted therapeutic option. Future research should focus on clinical translation aspects, including human trials, to fully assess the efficacy and safety of this innovative therapy. The successful adaptation of SPCSs for human use could significantly impact the management of thromboses, reducing the burden of this prevalent condition on healthcare systems worldwide.

In the evolving field of thrombotic disease treatment, the development of innovative drug-delivery systems is paramount. Among these, the deployment of external forces to drive micromotors for precise and efficient drug delivery represents a significant advancement. While our study introduces a groundbreaking approach utilizing SPCSs for targeted thrombolysis, the domain of micromotors is vast and includes various other mechanisms, notably US-driven micromotors.

US-driven micromotors, a method characterized by its deep tissue penetration capabilities, have emerged as a compelling alternative for targeted drug delivery. This technique leverages the mechanical energy of US waves to propel micromotors, enabling them to navigate through biological fluids and overcome physiological barriers to deliver therapeutic agents directly to thrombotic sites. The work of Prof. Daniel Ahmed’s team at ETH Zurich has been instrumental in advancing this field, showcasing the potential of US-driven micromotors. Their study highlighted the innovative design and application of these micromotors for enhanced drug-delivery efficiency [72]. Additionally, their recent works [73, 74] provide insights into the dynamic interactions of micromotors in biological environments, paving the way for new therapeutic modalities. A comprehensive review [75] offers a holistic view of the progress and challenges in the micromotor domain, suggesting future directions for research and application. Lastly, their investigation into the nanoscale dynamics of micromotors [76, 77] demonstrates the intricate mechanisms of action that underpin the functionality of these devices.

The advantages of US-driven micromotors are manifold. Their ability to penetrate several centimeters into human tissue stands out as a significant benefit, allowing for the delivery of therapeutic agents deep within the body without invasive procedures. This characteristic is particularly advantageous for treating vascular lesions located beyond the superficial layers of tissue. Moreover, the non-invasive nature of US propulsion minimizes patient discomfort and reduces the risk of infection. However, there are challenges to consider. In practical scenarios, predicting the precise location of a thrombus lesion poses a significant challenge for US applications. The precise control of micromotor movement under US propulsion can be complex, requiring sophisticated imaging and navigation systems to ensure accurate drug delivery. Additionally, the potential for acoustic damage to surrounding tissues necessitates careful optimization of US parameters to balance efficacy and safety. In contrast, the magnetic hyperthermia-driven approach of our SPCSs exploits the innate properties of sperm cells and magnetic RBCs upon AMF application to navigate and dissolve thrombi. This method leverages biological mechanisms for propulsion and drug release, presenting a novel strategy for thrombolytic therapy. While our study focused on the innovative use of spermatozoon-propelled cellular submarines for targeted drug delivery, the field of micromotors is diverse, with US-driven devices offering a complementary approach. The works of Prof. Daniel Ahmed and his team provide valuable insights into this area, highlighting the potential of US-driven micromotors in overcoming the limitations of current thrombolytic therapies. As we advance, the integration of these technologies may pave the way for a new era in treating thrombotic diseases, offering more efficient, safer, and less invasive options for patients worldwide.

This study explored the utilization of magnetically driven propulsion for drug delivery, a method that harnesses magnetic fields to navigate therapeutic agents to targeted sites within the body. This approach, while innovative, demands a comprehensive analysis to fully understand its implications, benefits, and potential drawbacks within the context of thrombotic disease treatment. The primary advantage of utilizing a magnetic field in drug delivery lies in the precision and specificity it offers [78]. Magnetic navigation allows for the directed movement of therapeutic agents to specific sites, minimizing systemic side effects and maximizing therapeutic efficacies [79]. This precision is particularly vital in treating thrombotic diseases, where targeted thrombolysis is crucial for effective treatment without harming surrounding tissues. Magnetically driven systems, such as iron oxide-based nanomaterials [80], leverage magnetic hyperthermia to facilitate the deep penetration of therapeutic agents into thrombi. This ability to overcome physiological barriers and deliver drugs directly to clot sites can significantly enhance treatment outcomes. The application of an AMF can trigger the release of encapsulated drugs from the carrier system [80]. This controlled-release mechanism ensures that drugs are only released upon reaching the target site, thus preserving their efficacy and reducing the likelihood of premature degradation.

The use of magnetic nanoparticles, particularly those based on Fe_3_O_4_, raises concerns regarding biocompatibility and toxicity. There is a risk of inducing oxidative stress and toxicity in the thrombus area due to the potential for the Fenton reaction to occur, which produces highly toxic reactive hydroxyl radicals [81]. However, our approach mitigates this risk by utilizing the magnetic properties of endogenous RBCs which accumulate within the thrombus, avoiding the introduction of additional innate magnetic materials. The successful application of magnetically driven drug-delivery systems requires sophisticated equipment to generate and control magnetic fields, which may limit accessibility and increase treatment costs. In practical settings, accurately predicting the exact location of a thrombus lesion presents a considerable challenge for effectively guiding and applying precise magnetic navigation [79]. This difficulty significantly impacts the strategic deployment of magnetic guidance systems, demanding enhanced methodologies for pinpointing thrombus sites to optimize therapeutic outcomes. Our innovative strategy harnesses SPCSs equipped with rheotaxis-based navigation to precisely target thrombus clots. It capitalizes on the innate magnetic properties of RBCs, which are naturally concentrated within thrombi. When these RBCs are exposed to an AMF, a magnetocaloric effect is generated. This approach eliminates the necessity for external magnetic materials, instead utilizing the body’s components to facilitate targeted drug delivery and thrombolysis. This method significantly minimizes the risk of toxicity, presenting a safer and more efficient therapeutic solution.

SPCSs exhibit remarkable motility and are capable of navigating toward and penetrating thrombi, where they release encapsulated drugs in response to magnetic hyperthermia. This process not only ensures targeted drug delivery but also stimulates macrophage infiltration and the production of M2 anti-inflammatory EVs within the thrombus. This multifaceted therapeutic action addresses the complexities of thrombotic diseases, offering a promising avenue for treatment that combines the benefits of magnetically driven drug delivery with the biological advantages of sperm cell propulsion and macrophage modulation.

The use of sperm cells for propulsion in the development of microcellular submarines (SPCSs) for drug delivery, particularly for thrombolytic and antithrombotic purposes, is based on several unique characteristics of spermatozoa that make them highly suitable for navigating and penetrating biological environments, especially for targeted therapeutic applications. Sperm cells are naturally motile and have the inherent ability to swim through fluid environments. This motility is powered by a flagellum, a tail-like structure that enables sperm to navigate and move in a directed manner towards the egg in reproductive processes [82]. This natural propulsion mechanism is highly efficient for navigating through viscous environments, such as those found within the in vivo milieu. One of the significant advantages of using sperm cells is their autonomous propulsion capability [83], eliminating the need for external power sources or additional equipment for movement. This autonomy ensures that the drug-delivery system can effectively operate in the body without external intervention. Moreover, sperm cells are biologically compatible and biodegradable, reducing the risk of adverse reactions or toxicity, which can be a concern with synthetic micromotors or externally powered systems. Sperm cells possess the ability to penetrate biological barriers [84], a trait crucial for fertilization. This characteristic is harnessed in SPCSs to enable the system to cross blood clot barriers and deliver therapeutic agents directly to thrombosis sites. The natural rheotaxis (movement against fluid flow) exhibited by sperm enhances their ability to home in on and penetrate clots, ensuring targeted delivery of drugs. While additional equipment might adjust the magnitude of propulsion, the use of sperm cells offers a delicate balance between sufficient propulsive force and the need for minimally invasive and biocompatible delivery vehicles. The propulsive force provided by sperm is adequate to navigate the microvascular environment and naturally penetrate clots, which are key requirements for thrombolytic therapy. Using sperm cells potentially avoids adverse effects of complications associated with mechanical propulsion systems, such as the need for external control mechanisms, toxicity concerns, or the risk of mechanical failure. Although the propulsion power of sperm cells may seem less complex than mechanical systems, their biological properties, including the natural ability to actively seek out and biocompatibly penetrate targeted areas, could lead to high delivery efficiency in specific applications like thrombus dissolution. Thus, the use of sperm cells in creating SPCSs leverages their natural propulsion and penetration abilities for targeted drug delivery, especially for thrombolytic purposes. The choice of sperm cells addresses concerns around biocompatibility, autonomy, and the ability to effectively navigate and penetrate biological barriers, making them a compelling option for innovative therapeutic biomaterial delivery systems.

This innovative approach leverages the self-propulsion capabilities of sperm cells for targeted drug delivery, offering advantages such as enhanced biocompatibility, extended lifespan, and improved tissue penetration, thus ensuring superior localization of therapeutic agents at lesion sites. Foundational research [85] highlighted the promising potential of drug-loaded sperm cells, equipped with micro/nanomotors, for precise navigation to target areas. Distinct from conventional nanomaterials, micro/nanorobots (MNRs) are capable of self-propulsion, converting ambient energy into mechanical motion. This attribute enables them to overcome limitations imposed by irregular Brownian motion and a low Reynolds number, facilitating their autonomous passage through dense biological barriers [86].

In particular, sperm-based biohybrid MNRs exhibit remarkable biological properties that are advantageous for medical applications. Their robust motility allows for effective navigation through viscous biological fluids, coupled with ample capacity for carrying drugs or imaging agents. Moreover, specific membrane components confer protection on loaded drugs from immune clearance, while their acrosomal structure promotes cell fusion, enhancing drug uptake by target cells. This is exemplified by a magnetic tetrapod-like microtube coupled with drug-loaded sperm, enabling precise delivery to tumor sites. Upon contact with a tumor spheroid, the sperm penetrates and fuses with cancer cells, facilitating targeted drug release [32]. These sperm-biohybrid MNRs hold significant promise for cancer treatment and other reproductive system diseases. Our research further demonstrates their potential in clot-penetration experiments, showing that administered SPCSs can effectively penetrate clots, thereby enhancing thrombolytic efficacy (Figs. 3 and 6).

The innovative approach discussed in this study, focusing on deploying SPCSs carrying anticoagulant drugs for treating thrombotic diseases, introduces a novel paradigm in the realm of thrombolytic therapies. This method leverages the inherent motility and viability of sperm cells, encapsulated within a microcellular framework, to navigate and penetrate thrombi, delivering therapeutic agents directly to clot sites. Enhanced by magnetic hyperthermia, this strategy not only promises targeted drug release but also capitalizes on the anti-inflammatory potential of treatment, demonstrating a significant in vivo thrombolytic efficiency.

The current landscape of thrombolytic therapies primarily includes the administration of drugs like tissue plasminogen activators (tPAs) and Uk, which are effective in dissolving blood clots but suffer from limitations such as short half-lives, requiring repeated high-dose administrations, and the risk of systemic bleeding due to poor biodistribution [7, 8]. Moreover, existing mechanical, thermal, and US methods, while promising, are restricted by their penetration depth and potential tissue damage [61–63]. In contrast, the SPCS system, employing magnetothermal effects facilitated by the accumulation of magnetic RBCs at the thrombus site, offers a depth-independent, targeted approach that mitigates the aforementioned limitations. This system ensures a higher specificity and efficiency in drug delivery and also minimizes side effects associated with systemic dissemination of thrombolytic agents. The underlying mechanisms contributing to the observed improvements in vascular blood flow and animal behavior post-treatment with SPCSs involve multifaceted therapeutic actions: SPCSs utilize the motility of sperm cells to navigate through the vascular system, specifically accumulating at sites of thrombi. This targeted approach ensures that therapeutic agents are released precisely where needed, enhancing the efficacy of thrombolysis [32]. The application of an AMF induces mild hyperthermia in thrombus areas rich in magnetic RBCs. This localized heating effect aids in the release of encapsulated drugs from the SPCSs and also promotes fibrinolysis, contributing to clot dissolution [68].

The heat generated by magnetic hyperthermia encourages the polarization of macrophages towards an anti-inflammatory M2 phenotype within the thrombus microenvironment. M2 macrophages, in turn, release EVs and express heat shock proteins (HSP70), which play crucial roles in reducing vascular inflammation, protecting endothelial cells, and preventing subsequent ischemic injury [31, 51–54]. By delivering a combination of antithrombotic and fibrinolytic agents directly to thrombi, while concurrently mitigating inflammation and facilitating endothelial recovery, the proposed system [32] fosters an environment conducive to vascular healing and restoration of normal blood flow. In addressing the query regarding the exploration of mechanisms behind the drug’s effects on vascular healing and functional recovery, it is evident that the study’s multifaceted approach encompasses not only the physical elimination of clots but also the biological restoration of vascular health. This comprehensive strategy, leveraging both targeted drug delivery and modulation of the immune response within the thrombus microenvironment, signifies a significant advancement over traditional thrombolytic therapies. The proposed SPCS system, with its innovative utilization of spermatozoon propulsion and magnetic hyperthermia, presents a promising alternative to conventional thrombolytic therapies. Its ability to provide targeted, efficient, and safe thrombolysis, coupled with its potential to mitigate ischemic damage, recover function, and promote vascular healing, underscores the need for further research and development in this direction. Future studies exploring the clinical applicability of this approach will be pivotal in harnessing its full therapeutic potential.

In addressing thrombotic cardiovascular diseases, a prevalent factor contributing to physical impairment and mortality, contemporary therapeutic strategies emphasize thrombolysis and ischemia mitigation. This study introduces an innovative cellular-cloaked SPCS, featuring multimodal motifs for integrating nano-assembled thrombolytics with immunomodulatory capabilities derived from innate magnetic hyperthermia. Utilizing rheotaxis-based navigation, these SPCSs home to and traverse clot barriers, culminating in their accumulation in ischemic vascular organs. Upon encountering thrombus magnetic RBC-driven magnetic hyperthermia, the thrombolytic motif activates, demonstrating potent thrombolytic activity and ischemic tissue amelioration in a murine model. This approach not only offers both thrombolytic and ischemia-mitigating effects but also suggests its extended bloodstream half-life and thrombus-targeting capability, heralding promise as an innovative therapeutic agent for enhancing efficacy in thrombosis management.

This innovative outlined approach significantly diverges from traditional thrombolytic therapies, which primarily rely on systemic administration of thrombolytic agents like tissue tPAs and Uk. While effective, these agents often necessitate repeated high-dose administration due to their short half-lives, potentially leading to adverse effects such as excessive bleeding [87]. Furthermore, the challenge of ensuring these agents’ effective delivery and penetration into compact fibrin networks of blood clots often limits their efficacy. In contrast, the SPCS system leverages cellular motility and targeted delivery, coupled with the unique utilization of magnetic hyperthermia, to achieve enhanced penetration and localized drug release within the thrombus, significantly minimizing the risks of systemic toxicity.

The integration of SPCSs for targeted drug delivery, thrombolysis, and cardiovascular regenerative medicine represents a notable advancement over conventional methodologies [32, 88]. It capitalizes on the rheotactic and innate magnetic properties of specific bodily components, such as RBCs in thrombi, to more effectively navigate towards and penetrate thrombi. This approach circumvents the need for external magnetic RBCs, leveraging endogenous cellular mechanisms for a more precise and less invasive intervention. By employing rheotaxis-based navigation and AMF-magnetic hyperthermia, this method significantly enhances the targeted delivery and efficacy of thrombolytic therapies, offering a promising solution to the limitations of current treatments. Overall, this study presents a groundbreaking advancement in the treatment of thrombotic diseases, demonstrating superior efficacy and precision in targeting thrombi. The SPCSs’ unique design, combining rheotaxis-based navigation with innate magnetic hyperthermia, offers a novel therapeutic modality with significant potential to improve patient outcomes in thrombosis management. The comparison with existing therapies underscores the innovative aspects of this approach, highlighting its ability to overcome the limitations of traditional thrombolytic agents and providing a foundation for future clinical applications and research in the field of thrombosis treatment. This analysis elucidates the advantages and addresses potential limitations, paving the way for further exploration and development of this promising therapeutic strategy in the battle against thrombotic cardiovascular diseases.

To address the potential safety concerns and limitations associated with using cellular-cloaked SPCS, it is crucial to consider a balanced valuation that weighs the novelty of the approach against practical risks and potential issues. This assessment is essential for providing a realistic view of the therapy’s applicability in clinical settings. The primary safety concerns with cellular-cloaked SPCS involve the unpredictability of in vivo navigation and the possibility of unintended interactions within the body [89, 90]. The propulsion mechanism of sperm cells, while innovative, may encounter navigational challenges in the complex and variable environment of human blood vessels. Moreover, the immune response to foreign cells, even if cloaked, could lead to inflammation or immune rejection. Furthermore, the long-term fate of these cellular constructs within the body should be studied in the future.

Male infertility, stemming from genetic abnormalities, endocrine disorders, inflammation, and exposure to toxic substances or gonadotoxic treatments, significantly impacts life quality for affected individuals. Consequently, recent research has focused on preserving and restoring fertility. A widely adopted practice involves biobanking tissue from testicular biopsies, securing a future source of sperm-related cells. Another promising method is the in vitro generation of haploid male germ cells. The ability of sperm-related cells to differentiate into sperm via testicular tissue transplantation, sperm cell therapy, and in vitro or ex vivo spermatogenesis positions them as prime candidates for in vivo fertility restoration. Successful transplantation of SSCs or testicular tissue to revive spermatogenesis and facilitate embryo creation has been documented in nonhuman mammals. While results from human trials are pending, this approach is anticipated to soon gain clinical acceptance for human applications [91]. In related techniques, in vitro spermatogenesis represents a pivotal advancement in treating male fertility issues such as sperm deformity and azoospermia [92, 93]. This technique holds vast potential across numerous clinical domains, not only for restoring fertility but also for investigating the cellular processes involved in male germ cell development. Furthermore, it plays a critical role in pioneering new methodologies for generating male germ cells in vitro, offering hope and new possibilities for reproductive medicine advancements.

Additionally, advancements in regenerative medicine that involve cultivating tissues or cells on organic or synthetic scaffolds infused with bioactive molecules are gaining momentum. These innovative techniques are currently at various stages of experimental research and clinical trials, promising a new era of solutions for male infertility. Translating spermatozoon-driven approaches to human clinical applications also raises significant ethical considerations. The use of human or animal sperm cells in medical treatments may encounter ethical objections based on cultural, religious, or personal beliefs. Additionally, the manipulation of reproductive cells for therapeutic purposes must be carefully regulated to avoid ethical misuse and ensure that such technologies are not applied for non-therapeutic enhancements or other controversial applications.

Despite these concerns, the potential clinical benefits of using SPCS for targeted drug delivery and localized treatment could be substantial [32, 35–37]. For instance, targeted hyperthermia has been shown to effectively enhance drug delivery and activate therapeutic agents within localized areas, reducing systemic toxicity. SPCS could potentially enhance this effect by providing direct and controlled delivery of heat and therapeutic agents to specific sites, such as thrombus or tumor tissues.

To advance the clinical applicability of this technology, rigorous preclinical and clinical trials are necessary to thoroughly assess safety and efficacy. Ethical considerations [94] can be addressed by engaging with ethical boards, regulatory agencies, and public stakeholders early in the development process to ensure transparency and public trust. Additionally, developing clear guidelines and protocols for the use of reproductive cells in medical treatments will be crucial. While the use of cellular-cloaked SPCS in medicine is an exciting prospect with considerable potential, it also presents significant challenges and risks that must be carefully managed through rigorous scientific validation and ethical consideration. Addressing these issues thoughtfully will be essential for the successful translation of spermatozoon-driven therapies from the laboratory to the clinic, ensuring that such innovations are both safe and socially acceptable.

## Conclusions

The study concludes by proposing a novel and comprehensive strategy for managing thrombi and providing encouraging treatment options for thrombotic illnesses. The application of DIP-SPCS-P6/Uk/PRO NP in conjunction with mild hyperthermia created by AMF has suggested to be remarkably effective in thrombolysis. In light of their preference for higher viscosity, the study indicated SPCSs’ capacity to home to thrombus environments. DIP-SPCS-P6/Uk/PRO NPs also demonstrated controlled drug delivery and retention inside thrombi, highlighting their potential as an effective and targeted drug-carrier system. The study also demonstrated how mild hyperthermia facilitated drug release and influenced macrophage polarization toward an anti-inflammatory M2 state, which has been linkedto protective effects against vascular injury. The results offer strong evidence that the magnetocaloric effect—which produces localized hyperthermia—is made possible by the presence of magnetic red blood cells within thrombi. This effect could play a major role in both the long-term suppression of thrombus recurrence and the dissolution of thrombi. Furthermore, the study demonstrated a decrease in thrombi’s vascular inflammation after DIP-SPCS-P6/Uk/PRO NPs were administered in conjunction with AMF-induced hyperthermia. In summary, this research provides a thorough strategy for managing thrombi, promoting thrombolysis, reducing inflammation, and mitigating ischemic damage. The results that are presented have important ramifications for the creation of non-invasive treatment approaches and help to advance creative approaches for managing thrombi.

### Electronic supplementary material

Below is the link to the electronic supplementary material.

Supplementary Material 1

## Data Availability

No datasets were generated or analysed during the current study.
