# Supplementary material for: Spermatozoon-propelled microcellular submarines combining innate magnetic hyperthermia with derived nanotherapies for thrombolysis and ischemia mitigation
Source: J Nanobiotechnology. 2024 Aug 8;22:470. doi: 10.1186/s12951-024-02716-w (PMC11308583; doi:10.1186/s12951-024-02716-w)
Supplement: Supplementary file 1 — Supplementary Material 1 [file 12951_2024_2716_MOESM1_ESM.docx]

**Supporting Information**

**Fig. S1** Spectroscopic data depicting conjugation of fluorescent CR110-conjugated hirudin peptide (P6).


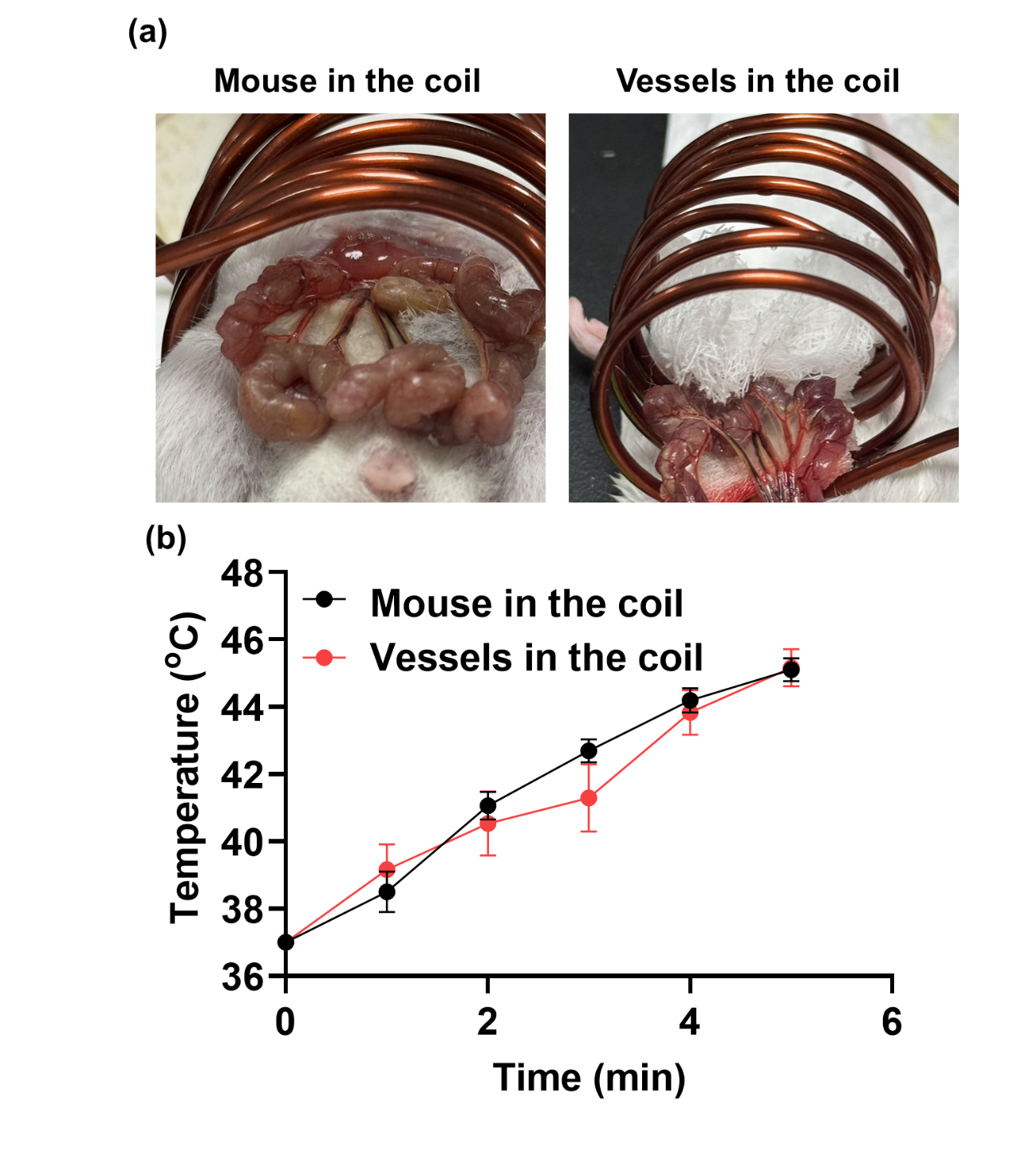


**Fig. S2** (a) Photographic and (b) in vivo temperature data (normal vessel- and thrombus vessel-bearing animals treated with an alternating magnetic field (AMF)).
